# Modulation of snow reflectance and snowmelt from Central Asian glaciers by anthropogenic black carbon

**DOI:** 10.1038/srep40501

**Published:** 2017-01-12

**Authors:** Julia Schmale, Mark Flanner, Shichang Kang, Michael Sprenger, Qianggong Zhang, Junming Guo, Yang Li, Margit Schwikowski, Daniel Farinotti

**Affiliations:** 1 Institute for Advanced Sustainability Studies, D-14467 Potsdam, Germany; 2 Paul Scherrer Institute, CH-5232 Villigen, Switzerland; 3 Department of Climate and Space Sciences and Engineering, University of Michigan, Ann Arbor, MI 48109-2143, USA; 4 State Key Laboratory of Cryospheric Sciences, Cold and Arid Regions Environmental and Engineering Research Institute, Chinese Academy of Sciences, 730000 Lanzhou, China; 5 Chinese Academy of Science Center for Excellence in Tibetan Plateau Earth Sciences, 100101 Beijing, China; 6 Institute for Atmospheric and Climate Science, Swiss Federal Institute of Technology, CH-8092 Zurich, Switzerland; 7 Key Laboratory of Tibetan Environment Changes and Land Surface Processes, Institute of Tibetan Plateau Research, Chinese Academy of Sciences, 100101 Beijing, China; 8 Swiss Federal Institute for Forest, Snow and Landscape Research, WSL, CH-8903 Birmensdorf, Switzerland; 9 GFZ German Research Centre for Geosciences, Section 5.4 - Hydrology, D-14473 Potsdam, Germany; 10 Laboratory of Hydraulics, Hydrology and Glaciology (VAW), Swiss Federal Institute of Technology, CH-8092 Zurich, Switzerland

## Abstract

Deposited mineral dust and black carbon are known to reduce the albedo of snow and enhance melt. Here we estimate the contribution of anthropogenic black carbon (BC) to snowmelt in glacier accumulation zones of Central Asia based on *in-situ* measurements and modelling. Source apportionment suggests that more than 94% of the BC is emitted from mostly regional anthropogenic sources while the remaining contribution comes from natural biomass burning. Even though the annual deposition flux of mineral dust can be up to 20 times higher than that of BC, we find that anthropogenic BC causes the majority (60% on average) of snow darkening. This leads to summer snowmelt rate increases of up to 6.3% (7 cm a^−1^) on glaciers in three different mountain environments in Kyrgyzstan, based on albedo reduction and snowmelt models.

Black carbon (BC) has recently received significant attention due to its short-term climate warming effects^1^,^2^. In addition to the direct and aerosol-cloud interaction effects, BC can exert radiative forcing when deposited on glaciers and snow through albedo reduction^3^. This effect can become significant in regions with high BC deposition^4^ and can lead to enhanced (glacier) melt. The effect on snowmelt through BC and other light absorbing impurities (LAI) such as mineral dust depends on a variety of factors, including the absorptivity of the LAI, snow grain size, solar zenith angle, and cloud cover^5^. Additional factors, particularly relevant for Central Asia where glaciers experience year-round snowfall^6^, are the temporal patterns of LAI deposition, and summer temperatures. New snowfall can slow this process by refreshing snow albedo, but after melting, impurities are exposed again. Insoluble LAI such as BC and mineral dust are also retained at the glacier surface during melt^7^. Their surface concentration can hence increase as fractions of LAI from several melted snow layers are combined. Also additional input from dry and wet deposition - including successive melting - can occur. Despite the potentially large effect, only few studies have estimated the enhanced melt rates due to LAI in snow and ice (see ref. 8 for a recent review). The two areas that have received most attention are the western United States^9^,^10^,^11^,^12^ and the Hindu-Kush-Himalayan region^13^,^14^,^15^. While the majority of the studies focus on either mineral dust or BC, only few have attempted to compare the impact of various LAIs^9^,^15^.

In the context of snow and glacier evolution, so far, studies in Central Asia have focused mostly on climate change effects. For the Tien Shan, Central Asian’s largest mountain ranges, glaciers are anticipated to lose up to 50% of their mass by 2050 (ref. 6). This is detrimental since most of the local population depends on snow and glacier meltwater supply^16^, and densely populated areas near lower-lying mountain ranges are particularly vulnerable^17^. In addition to increasing temperatures, glaciers and mountain snow are also affected by deposition of mineral dust from the Central Asian dust belt, and BC from anthropogenic activities and wild fires^18^ (Fig. 1). However, the importance and provenance of these LAI contributions are unclear. Here, we address their contribution to snow albedo reduction and investigate whether LAI impacts of anthropogenic or natural origin dominate. We analyse their concentration in snow, provenance, anthropogenic contribution, albedo reduction, and implications for snowmelt in glacier accumulation zones. Definitions of LAIs in this work are: (a) BC refers to particles defined within emission inventories as BC. For field and modelling studies, we use BC as a general term and specify whether elemental carbon (EC) or any other specific BC type has been used. Natural BC includes emissions from wildfires only. Anthropogenic BC represents all other BC emissions, including from domestic biomass burning. (b) Mineral dust is defined as non-water soluble, non-combustible residue when filtering melted snow through a filter with a pore size of 0.45 μm (see Methods). (c) LAIs are the sum of mineral dust, anthropogenic and natural BC.

## Results

### Impurity concentrations in snow

**Figure 1:**
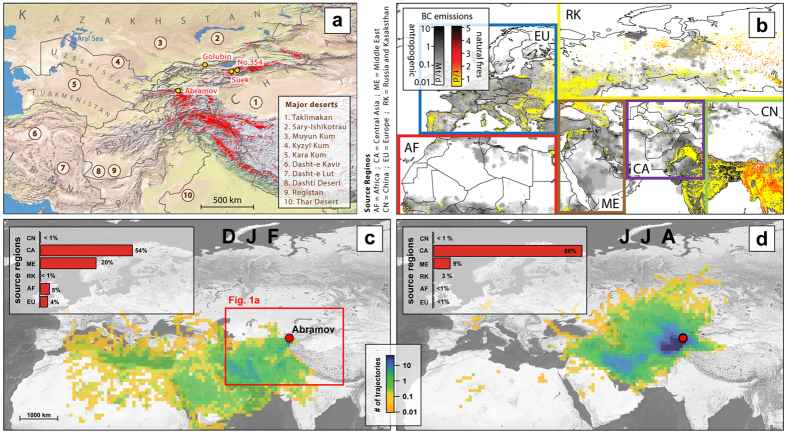
Study area, BC emissions, and footprint analysis. (**a**) Sample sites (yellow dots), glacierized areas (red), major desert surrounding Central Asia (brown areas, circled numbers), and vegetated zones (green). Golubin, Suek and No. 354 are located in the Tien Shan, Abramov in the Pamirs. (**b**) Eclipse V5 annual anthropogenic (grey shading) and FINN v1.5 natural fire (coloured dots) BC emissions. The inventories refer to 2010 and 2013, respectively. Boxes define the applied regional classification. (**c**) and (**d**), examples for boundary layer 5-day back trajectory footprints for winter (DJF) and summer (JJA) 2013 for Abramov glacier. Inlays depict the regional fractional contribution of air masses. The map in panel (a) was created with Quantum GIS v.2.6 (http://www.qgis.org/de/site/) using Natural Earth I raster maps (http://www.naturalearthdata.com/downloads/10m-raster-data/10m-) and world borders (http://thematicmapping.org/downloads/world_borders.php), glacier locations were taken from the Randolph Glacier Inventory^61^ (http://www.glims.org/RGI/). Background maps in panels (c) and (d) are the same as in (a) without borders. The emissions and back trajectory footprints were created with Igor Pro v.6 (https://www.wavemetrics.com/) and overlaid.

To estimate the contribution of anthropogenic BC to snowmelt on glaciers, we perform a series of analyses based on measurements of EC and mineral dust concentrations in snow (Methods). As recommended by ref. 19 in the context of emission inventories and modelling, we analysed samples for EC but refer to it as BC, which is used as a general term. A total of 226 samples from 13 snow pits were taken from annually accumulated snow representing the period between August 2012 and August 2013 (samples from summer 2013) and the period from August 2013 to August 2014 (samples from summer 2014) on four different glaciers in Kyrgyzstan: Abramov, Suek Zapadniy (hereafter referred to as Suek), No. 354, and Golubin (Fig. 1a and Supplementary Material (SM) Sec. 1). Concentrations of EC and mineral dust were determined by a thermal-optical reflectance method^20^ and gravimetry^21^, respectively. Concentrations of oxygen isotopes, heavy metals, and Fe were also determined (Methods).

**Figure 2:**
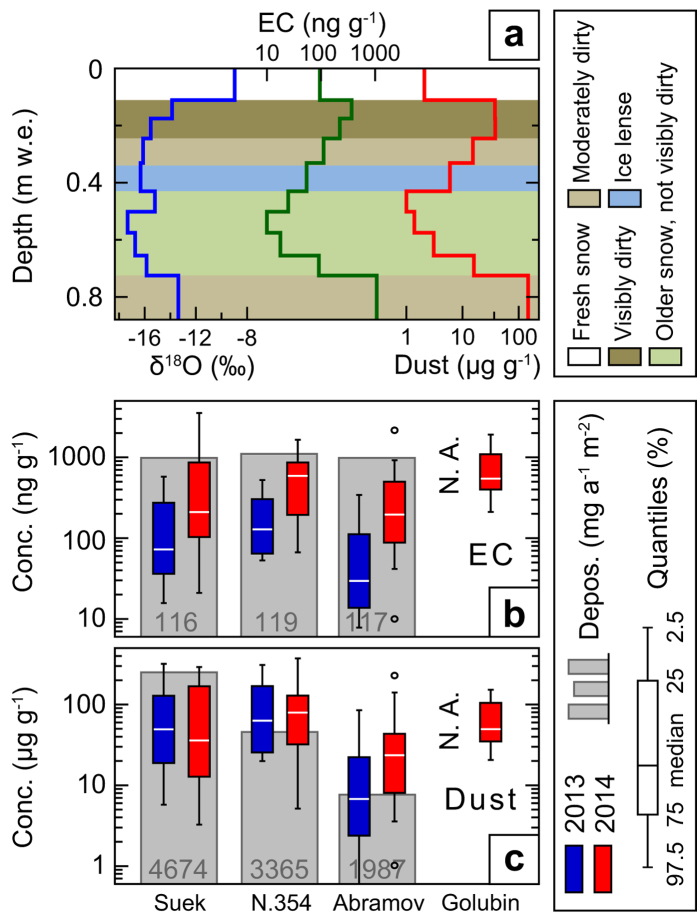
Burden of impurities. (**a**) Example of individual snow pit data for the isotopic composition, and EC and mineral dust concentrations (Abramov 1a 2014 shown here, see also SM Section 3, Table S4–S6). The shading indicates snow characteristics. (**b,c)** Annual concentrations of (**b**) EC and (**c**) mineral dust for all snow pits per glacier and year. Grey bars represent the average annual deposition between 2012 and 2014. For Golubin, 2013 values are not available (N.A.).

We find EC concentrations from all individual samples to vary between 70 and 502 ng g^−1^ (interquartile range, IQR), reflecting the large variability of deposited LAI in individual snow pits, glaciers, and years (Fig. 2a,b). Patterns of the annual concentration profiles (SM Section 3, Table S4–S6) are similar on all glaciers, with low concentrations in winter and high concentrations in summer. Generally, glaciers in the Tien Shan (Golubin, No. 354, Suek) show higher concentrations than the one in the Northern Pamirs (Abramov) while the annual deposition flux of EC is very similar (Fig. 2b). On Abramov, for example, higher snow accumulation leads to more ‘diluted’ EC concentrations than for the Tien Shan glaciers, but to a similar annual EC burden. Compared to measurements from Muztagh Ata, further South in the Pamirs, where EC concentrations ranged from 52–152 ng g^−1^ in a snow pit, concentrations on Abramov are higher. The median EC concentrations on Suek and No. 354 (Fig. 2b) are similar to findings derived with similar analyses from the West Chinese Tien Shan^22^ where concentrations exceed 100 ng g^−1^ as well. Concentrations in the Tien Shan are highest for Golubin, likely due to its proximity (~35 km) to the capital Bishkek, and are within the range of snow contamination in industrial China^23^ where BC values of more than 1000 ng g^−1^ were found. The highest EC concentrations in each snow pit where found in layers that had undergone melting. These layers were either located at the surface or at the bottom of the snow pit representing the current or previous summer layer. Such accumulation of LAIs has been observed before in various locations^7^,^21^.

For dust, concentrations and deposition fluxes differ more strongly from site to site, but show less variability between the two years (Fig. 2c), even though variability can be substantial^24^. Concentrations are in the range of 2 to 167 μg g^−1^ (IQR) and are similar to recorded dust loads found in a firn core on Inilchek glacier^25^, East Kyrgyzstan, when using Fe as dust proxy. In our samples, Fe and dust correlate closely (R^2^ = 0.76, p < 0.01, slope of 0.0083 g Fe per g mineral dust). Average Fe concentrations on Inilchek are 550 ng g^−1^, while we find 390 ng g^−1^ with a likely lower extraction efficiency for Fe than in ref. 25. Compared to data obtained with the same extraction and measurement methods from a firn core from Fedchenko glacier^26^, further South in the Pamirs, our average Fe concentration is eight times higher, indicating that the Northern Pamirs and Tien Shan receive higher dust loads. Dust concentrations found in the Eastern Tien Shan in China are significantly higher on average with about 1000–3700 ng g^−1^ increasing from West to East^27^.

LAI deposition observations can depend strongly on the sampling location^28^,^29^. We selected various locations in the accumulation zone on flat glacier sections while maintaining distance from surrounding mountains and slopes. Areas with wind-blown snow were avoided as well. Considering surface samples only, we find an average concentration of EC (dust) of 57 ng g^−1^ ± 51% (11 μg g^−1^ ± 108%) in fresh snow, 175 ng g^−1^ ± 40% (46 μg g^−1^ ± 76%) in several days old snow, and 1110 ng g^−1^ ± 68% (200 μg g^−1^ ± 72%) in snow with melt forms (see section ‘Albedo reduction’ for snow age classification). Inter-annual variations of LAI concentrations in snow can depend on their emissions, atmospheric transport and deposition. While the two latter factors would have to be modelled with an atmospheric transport model for a multi-year comparison, we can compare available emission data to investigate inter-annual variability. Even though dust emissions can be highly episodic, emissions in Central Asia between 2012 and 2014 were not exceptional compared to the period 2000–2014 (ref. 24). Hence the samples are likely representative of the average long-term regional dust burden. Yearly data for anthropogenic BC emissions in Central Asia from the Regional Emission Inventory in Asia (REAS)^30^ indicate a growth rate of 2.8% per year during 2000–2008. This means that for 2012 to 2014, a slight increase in emissions was expected, however without large inter-annual variation. Moreover it must be noted that such an increase is likely not measurable in snow due to the variability of other atmospheric processes. For BC emissions from natural fires, which can also vary significantly between years, we find that emissions in 2012 to 2014 were about 20% lower than the average from 2003 to 2014 (see section ‘Origin and fractional contribution of anthropogenic black carbon’ for a detailed discussion).

### Provenance of air masses

We calculated the number of back-trajectories intersecting the boundary layer (“footprints” in the following), based on the Lagrangian analysis tool LAGRANTO^31^,^32^ to determine the regional origin of air masses affecting the sampling sites (Methods). Both the Tien Shan and the Pamirs are frequently influenced by westerly winds^33^, more so in winter than in the other seasons (Fig. 1c,d and SM Sec. 4). During spring and summer, the Siberian anticyclone induces air mass transport from higher latitudes. During spring and fall, the extratropical cyclones of the mid-latitudes are responsible for air mass transport from the East, in particular from the Taklimakan region. More than 70% of the air masses come from Central Asia during all seasons except winter. In winter, transport from the Middle East becomes more important (~30%), while from spring through autumn this is the case for air masses from China (except for Abramov). Hence, local sources and the Central Asian dust belt are the main contributors to mineral dust deposition on the glaciers. This is supported by a modelling study^18^ focusing on sources of airborne particulate matter in Kyrgyzstan that locates PM_2.5_ (particulate matter with a diameter smaller than 2.5 μm) dust sources mainly in Central Asia but also in Western China, Africa and the Middle East. Questions have arisen whether dust emitted from the dried Aral Sea basin would deposit in the Tien Shan and Pamirs^34^. Since elemental ratios in our samples are very different from ratios expected for Aral Sea mineral dust^35^, however, contributions from this regions are likely to be very small (SM Sec. 3.2).

### Origin and fractional contribution of anthropogenic black carbon

The contribution of anthropogenic BC is quantified by multiplying the Lagrangian footprints with monthly averages of anthropogenic emissions data from 2010 (ref. 36). A monthly averaged fire emission inventory^37^ is used for the time period 2012–2014 to derive BC contributions from natural fires. These emission inventories contain BC instead of EC emissions. However, BC can be used as a proxy for EC in this case, as the relevant information is the mass ratio of emitted BC from fires and anthropogenic activities. While the footprints consider BC as a passive tracer (that is wet or dry deposition during transport is not accounted for), they allow for an estimation of the relative source strengths. This is because air masses that originate from the same region at the same time will undergo the same atmospheric processes, including wet or dry deposition, independently on whether they carry anthropogenic and/or natural BC emissions. Our results agree well with those from a global model^38^ parameterizing deposition processes and using the same emission inventory (SM Sec. 6). According to the estimates based on our combined back trajectory and emission inventory, anthropogenic emissions are the dominant contributor, accounting for > 94% of the BC mass found at all glaciers in all seasons (SM Sec. 5, Fig. S7). Similar shares have been found in two modelling studies^18^,^38^ (SM Sec. 6). The most important source is Central Asia, which accounts for more than 50% in winter and about 70% during the rest of the year. Middle East emissions are important for Abramov year-round (~10%) while the other glaciers also receive BC from China (up to 10%) between March and November. These findings are in general agreement with a modelling study^39^ addressing the origin of BC depositions in the high Pamirs. In terms of source type, domestic and traffic activities are responsible for more than 60% of BC emissions affecting each considered glacier (SM Sec. 6). Note, however, that our approach of using 5-day back trajectories likely overestimates contributions from within short to medium distances (e.g., Central Asia, Middle East) compared to long distance source regions (e.g., Europe). A comparable modelling study^18^ finds about 10% anthropogenic BC contribution from Europe (>1% in this study) while another^39^ attributes much lower contributions to Europe. The high relevance of anthropogenic emissions for LAI concentrations is also supported by enrichment factors of up to 100 for heavy metals (SM Sec. 7). Natural fires contribute little (<6%) to BC concentrations, with the largest impact found during summer. Source regions include Central Asia but also the Middle East and Russia/Kazakhstan.

To estimate how representative BC fire emissions were between September 2012 and August 2014, we compared yearly (September to August) FINN v1.5 emissions over the period from 2003 to 2014 from the most relevant source regions (Central Asia, Middle East, Russia, and Kazakhstan). Between September 2012 and August 2014 (and also between September 2011 and August 2012), inventory emissions were 20% lower than on average over the whole period. Hence, our estimated contribution from natural biomass burning BC to total BC (<6%) could be slightly lower than in other years. Assuming average BC fire emissions, the contribution would be around 7%.

### Albedo reduction

**Figure 3:**
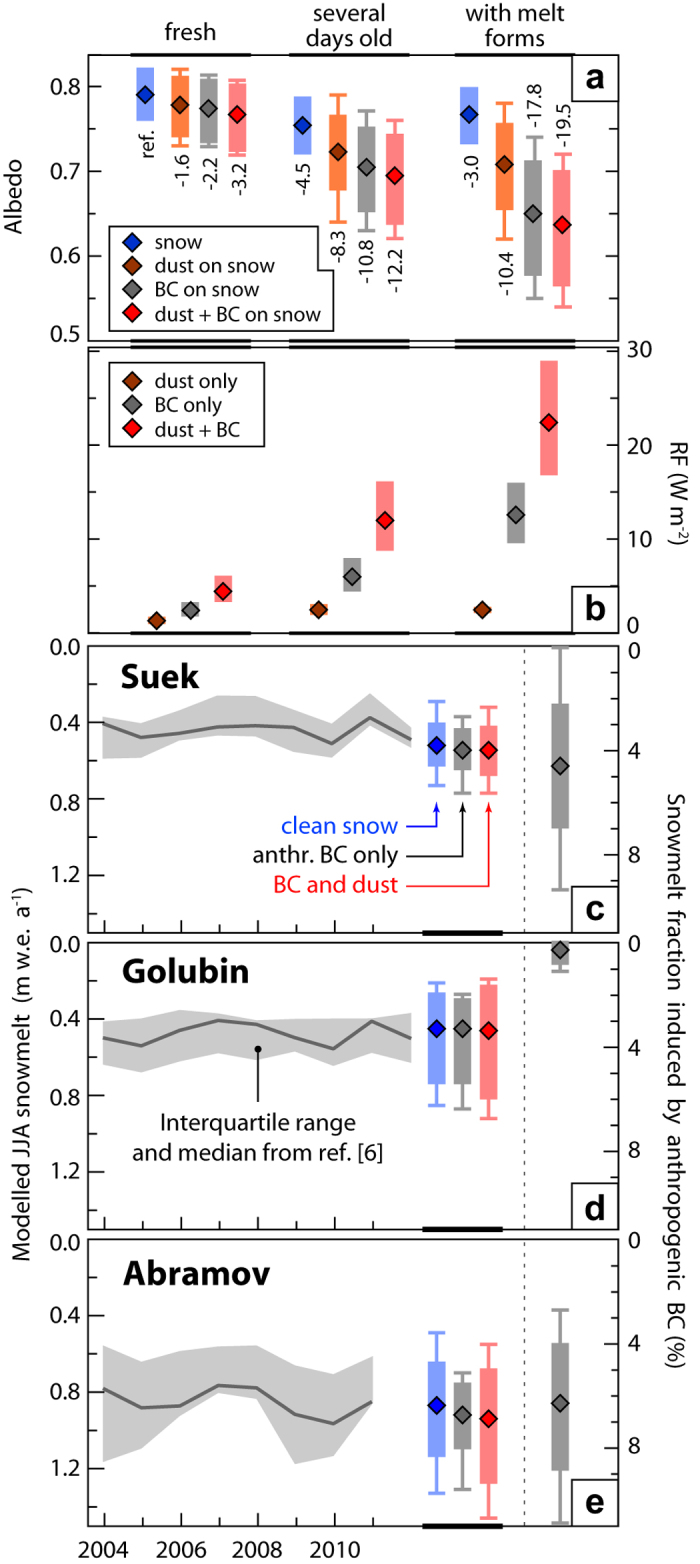
Effect of light absorbing impurities on albedo, radiative forcing and snowmelt. (**a**) Albedo reduction of snow due to aging only (blue) and impurities (other colours) relative to the clean, fresh snow (reference). The diamonds are the central estimate while the bars show the range of the snow grain size scenarios. In addition to the snow grain size effects, whiskers include the effect of varying LAI concentrations by a factor of 2. (**b**) Resulting all sky daily-mean radiative forcing (RF, diamonds and bars as above). (**c–e**) Left: comparison between JJA snowmelt time series from ref. 6 (grey band) and modelled 2013–2014 JJA snowmelt (squares); bars represent the meteorological variability range, whiskers the additional variability due to the extreme cases of albedo change as shown in (**a**); Right: snowmelt contribution from anthropogenic BC (diamonds are central estimates, bars (whiskers) are the propagated meteorological (total) variability). The central estimate of the melt contribution from anthropogenic BC (Fig. 3c–e right panels) is based on the central melt scenarios and the range is based on variability propagation from the largest scenario ranges (Fig. 3c–e left panels).

To calculate albedo change and surface radiative forcing (RF) from LAI, we use the multi-layer Snow-Ice-Aerosol-Radiative-Model^4^ (SNICAR) and apply it to each snow pit. Since optical characteristics of mineral dust can vary significantly by region, we did not use the SNICAR default dust optical properties, but used regionally specific data. Snow grain size was considered in low, central and high estimates and the model was run for all-sky conditions (including clouds and atmospheric aerosol layers) on the specific sampling days (Methods and SM Sec. 8). Uncertainties in the determination of LAI concentrations are also accounted for by deriving snow albedo for doubled (halved) contamination. We classify all snow pits according to surface snow types that are typical for summer conditions in the region: ‘fresh snow’ (≤1 day, 5 pits) with typically the lowest LAI concentrations; ‘several days old’ snow (>1 day, no melt forms, 3 pits) on which impurities accumulate likely through dry deposition^9^,^23^, representing mostly the second dirtiest snow layer per snow pit; and snow ‘with melt forms’ (5 pits) on which LAI are retained due to their insolubility^7^, mostly representing the layers with the highest LAI burden in each snow pit (SM Sec. 9, Table S10). The average snow albedo reduction and RF are shown in Fig. 3a and b as a function of surface snow type. The coloured bars reflect the sensitivity of the albedo reduction to the snow grain size (which has the largest effect) while the whiskers indicate the change in albedo with doubled (halved) LAI concentrations in addition. We find absolute albedo reductions between 3 and 19% for the combined effect of BC and mineral dust (central estimates). This is in the range of findings for the Himalayas^15^. Mean RF in August due to BC and mineral dust ranges between 5 and 23 W m^−2^ (central estimates) depending on snow type.

We find that BC and mineral dust are of similar but low (moderate) importance in fresh (several days old) snow. In snow with melt forms, however, LAI strongly reduce albedo (up to 19%). This is highly relevant since RCP 2.6 (8.5) scenarios anticipate average daily June-to-August (JJA) temperatures to rise about 1.5 (4.0) °C by 2050 (ref. 40) which would cause snow with melt forms to occur more often. Moreover, the effect of BC exceeds that of dust in all snow types (Fig. 3a). Albedo reduction from anthropogenic BC is dominant in almost all surface samples. On average, anthropogenic BC is responsible for 59 ± 9% of the albedo reduction caused by LAIs while dust and biomass burning BC account for the rest. The albedo reduction varies from 3.2% to 19.5% depending on the snow type (Fig. 3a). On Abramov, the anthropogenic BC contribution ranges from 48 to 77% (58% on average), on No. 354 from 47 to 74% (61%) and on Suek from 17 to 59% (57%). On Golubin only one surface sample was taken (60%). This observation is in contrast to studies in the Himalayas, where mineral dust was found to be more important^13^,^28^. The larger importance of anthropogenic BC (compared to mineral dust) on albedo reduction might be counter-intuitive since regional desert dust emissions result in 20 to 50 times higher annual mass depositions than EC (Fig. 2a,b). However, this can be explained by the low absorptivity of the dust. The latter owes to the low Fe content (0.83%) and regional iron oxide specificities^41^,^42^ (SM Sec. 8).

### Snow melt

Based on the LAI albedo change (Fig. 3a), we model snowmelt enhanced by LAI for different cases in which we vary meteorological parameters (SM Sec. 10). The JJA snowmelt rate for each glacier is calculated individually with and without LAI. The model is validated against the snowmelt rates from the 18 glaciological models used in ref. 6 (Fig. 3c–e). Note that most of the enhanced melt related to LAI is due to BC, of which an estimated 94% is of anthropogenic origin. The current contribution to snowmelt from anthropogenic BC (Fig. 3c–e) is largest on Abramov with a central estimate of 6.3%. This corresponds to a melt rate enhancement of 0.8 mm d^−1^, and is due to the larger snow covered area on the glacier and the less frequent fresh snowfall on Abramov as compared to Suek and No. 354. On these latter two glaciers the contribution is about 4.6%, with a melt rate enhancement of 0.4 mm d^−1^. The low (<1%) contribution on Golubin is related to the generally scarce summer snow covered area on this glacier^17^ mainly related to the high air temperatures which caused the equilibrium line altitude to move beyond the highest point of the glacier.

## Discussion and Conclusion

The analyses presented here refer to enhanced rates of snow melt only, and do not account for the same effect in glacier-ice melt. The melt rates stated above are, thus, only a lower bound for the total effect of LAIs. For the combined LAI impact on snow and ice melt, two effects are relevant: First, LAI also reduce the albedo of ice, although the effect is smaller than for snow due to the generally lower albedo of ice compared to snow^13^ unless there is a layer of cryoconite (a dark mixture of fine dust, small rocks, BC and microbiological material) which would significantly reduce the albedo; second, ice melt can be indirectly enhanced due to the presence of BC in snow, as the latter is likely to cause the snow to deplete earlier and, thus, to longer expose bare ice to direct solar radiation.

The chain of analyses from LAI concentration measurements to snowmelt rate contributions is subject to a variety of uncertainties. When determining the albedo reduction, potential under- or overestimation of LAI concentrations were conservatively accounted for by varying the concentrations by a factor of 2 (Methods, SM Section 2, 8, and 11). With regards to using EC mass instead of a BC type (refractory or equivalent, for example) as input to the SNICAR model, we do not introduce any additional uncertainty, because the model assumes a universal mass absorption cross section (7.5 m^2^ g^−1^) for its generic BC input requirement, and hence using EC mass is justified. The effect of snow grain size is generally larger than the uncertainty in LAI concentrations (compare whiskers-length and bar-range in Fig. 3a) and varies with the snow type. Given the possibility that we might underestimate the mass fraction of Fe (0.83%) by up to a factor of three in the snow samples (Methods), we performed the calculations of the albedo reduction also with 3% Fe content in mineral dust (SM Sec. 11). We find that BC would still remain the more important driver in the snow types ‘several days old’ and especially in snow ‘with melt forms’: In the former case, it is 1.03 times more important (not significant) than mineral dust compared to 1.29 times with 0.83% Fe, and in the latter case, the factor is 1.26 compared to 1.71 with 0.83% Fe.

We propagate the range in snow albedo reduction results to the melt calculations by using the extreme albedo values in addition to the central albedo value for each snow type. Furthermore, parameters such as the (a) temperature of the snow, (b) relative humidity, (c) wind speed, (d) ambient temperature, (e) number of snowfall events, and (f) snow covered area fraction on the glacier, can introduce both uncertainty in the results and variability over several years. We consider ‘(a)’ by calculating snowmelt at 0 °C and −5 °C, ‘(b–d)’ by accounting for sublimation, and ‘(d–f)’ by varying the corresponding observed variables by ± 25% (SM Sec. 10). From the combination of all variables, we show the range of the resulting 2013–2014 JJA snowmelt rates coming from the meteorological variability alone (coloured bars in Fig. 3c–e) and the extreme cases when accounting for both the meteorological and albedo variability (whiskers in Fig. 3c–e). The variability in the albedo (meteorological parameters) contributes about 20% (80%) to the estimated variability in snowmelt. This analysis suggests that the combined variability of meteorological parameters and snow albedo could be 3 times larger than the inter-annual variability indicated by the model results from ref. 6.

Despite the potentially large uncertainties as reflected by the variability in the results, our analyses show that snow on glaciers in the Central Tien Shan and Northern Pamirs is significantly affected by LAIs, and that the largest fraction of the effect is caused by anthropogenic BC. In particular, BC emissions have a larger effect than mineral dust, although the deposition rates for the latter are higher by at least one order of magnitude. The decreasing trend of dust emissions in Central Asia^24^, suggests that mineral dust might become even less important in the future while more BC might be deposited due to the projected increase in emissions^30^. Our results also suggest that LAIs have only a limited effect on glaciers that show high snowmelt rates already and where the equilibrium line altitude (ELA) is above the highest point of the glacier, such as for Golubin. For the other glaciers, where the ELA is expected to migrate upwards in a warming climate, the layers with very high impurity concentrations and hence the larges albedo reduction, which were observed to be typical for late summer due to the melting and freezing cycles, could become exposed thus making the glacier surface even darker. Similar observations have been made in the Himalayas^21^. The potential implications have yet to be quantified.

Since most of the deposited BC comes from regional anthropogenic emissions, Central Asia has a leverage to prevent rates of BC-enhanced snowmelt on glaciers to further increase. Reducing BC is easier than decreasing CO_2_ emissions^1^ – that would have a similar effect on snow and ice melt^43^. These are two additional arguments for regional action for more stringent control on air pollution while developing more long-term strategies for CO_2_ reduction. Central Asian countries are among the top ten nations globally that would benefit most from air pollution reduction in terms of avoiding regional temperature increase, premature deaths and crop losses^1^, and, in addition, action could help to slow the thawing of Central Asian glaciers.

## Methods

### Snow sampling and analyses

13 pits representing one year of snow accumulation were dug in summer 2013 and 2014, and a total of 226 samples were taken (SM Section 1). 500 ml samples were taken following the ‘dirty hands, clean hands’ protocol^44^ and stored in Nalgene^®^ bottles. After sampling, the bottles were stored frozen in the dark until shipment. Upon arrival, samples were processed immediately in a class 100 clean room. An aliquot of each sample, melted at room temperature, was analysed with a Picarro Liquid Water Analyzer (Wave Scan-Cavity Ring Down Spectrometer, L2130i), with a standard error of <0.05‰ for *δ*^18^O and <0.5‰ for δD. A fraction of each sample was filtered through quartz-fibre filters (Tissuquartz 2500QAT-UP 47 mm, 0.45 μm pore size, Pall) following the method described by refs 21, 27 and 45. The filters were weighed before and after filtration using a microbalance (accuracy of 0.1 mg). The difference between the two weights was used as a proxy for the mass of insoluble dust present on the filter. From the melted samples, particles in the size range of 0.57 to 387.15 μm equivalent spherical diameter were counted using an Accusizer 780A (ref. 27). The estimated total uncertainty for particle concentrations is <1% (including background counts and random counting error). For further details see SM Sec. 2.1.

0.526 cm^2^ punches of the filters were used for EC analysis. To avoid possible positive EC artefacts from the formation of CO_2_ from inorganic carbonates, the latter were removed before analysis by dripping 50 μl of 0.1 M HCl onto the sample three times^46^,^47^. A thermal-optical reflectance method for carbon analysis^20^, DRI^®^ Model 2001A OC/EC, was employed to measure the particulate organic and EC mass on the filters (measurement range: 0.2–750 μg C cm^−2^). Measurements followed the Interagency Monitoring of Protected Visual Environments (IMPROVE) thermal-optical reflectance protocol^48^. The analytical uncertainty is estimated to be 15% for EC^49^. Values reported here likely underestimate the EC concentration for two reasons: First, the retention of EC on the filter can be as low as 38% as reported by ref. 50 for rainwater. However, since carbon particles tend to agglomerate in snow samples^51^ the retention is likely higher in this case. Second, in case of high mineral dust loading, carbon and dust particles can agglomerate so that elemental carbon is only inefficiently burned and hence its concentration underestimated^52^. Further underestimation can occur if the mineral dust is highly absorptive^52^ which, however, is not the case for this set of samples.

The melted samples, digested with 1% nitric acid for 24 hours, were analysed for further elements including Fe by inductively coupled plasma-mass spectrometry (ICP-MS, X-7 Thermo Elemental) following the procedure in ref. 53. The measurement uncertainty was estimated to be <5% based on the relative standard deviation of repeated measurements. See SM Sec. 2.2 for further details. The method applied here for digestion is likely to underestimate the Fe concentration in mineral dust by a factor 1.5 to 5 (refs 54, 55, 56, 57). Given that we find an Fe fraction of roughly 1% and that the fraction in the upper continental crust is about 3% (ref. 58), a factor 3 seems plausible. See SM Sec. 11 for snowmelt implications.

### Back trajectory analyses

Back trajectory analyses were preformed using operational ECMWF analyses fields (0.25° x 0.25° resolution) with the Lagrangian analysis tool LAGRANTO^31^,^32^. Seasonal footprints were derived for Abramov, Suek (also representative for No. 354) and Golubin for the period from August 2012 to September 2014. Backward trajectories were launched every six hours between 1 August 2012 and 31 August 2014. Air parcel positions falling within the planetary boundary layer (PBL) were saved for all seasons (see Fig. 1c,d and SM Sec. 4) and for each glacier. This allowed the air mass origin to be classified into six regions (Fig. 1b). For the five-days back trajectories, trajectory divergence (or coherence) was taken into account by varying the release altitude (set to the altitude of the snow pits) in 50 hPa steps and shifting the starting positions by ± 0.05° in east-west and north-south directions. This resulted in an ensemble of 40 members per trajectory calculation. The method attributes more weight to coherent trajectory ensembles, i.e., when many of the 40 ensemble members leave simultaneously their footprint in the boundary layer. If, for instance, only one member of an ensemble contributes to the footprint, the ensemble’s impact on the footprint and the BC emission is accordingly smaller. For BC emissions from natural fires, the FINN v1.5 global fire emission inventory, speciated with the GEOS-chem mechanism^37^, was used. For this case, footprints include back trajectories touching down in the PBL and within 100 hPa above the PBL, thus taking into account the injection height of fire emissions in the region^59^.

### Snow albedo and melt calculation

As input to the SNICAR model, snow densities, the stratigraphy, measured EC, and gravimetrically determined mineral dust profiles were used from the full depth of each snow pit. Snow grain sizes were assigned based on the stratigraphy of qualitative snow type. In the central scenario, they ranged from 100 μm for fresh, clean snow, to 1500 μm for old, icy and dirty snow (SM Sec. 8, Table S9). Grain sizes were scaled up and down by a factor of two for the high and low scenarios. A dust particle size distribution of 1.85 μm geometric mean diameter by volume and a geometric standard deviation of σ_g_ = 2.0 was applied based on the available measurements (SM Sec. 8, Fig. S9). Volume fractions of hematite and goethite were fixed at 0.0014 and 0.0024, respectively. Fractions of other minerals were held in the same proportion as the “low hematite” scenario from ref. 60. Mie calculations were performed to derive one set of optical properties for dust, whilst optical properties of black carbon were identical to the hydrophobic BC properties used in ref. 4.

To estimate the snowmelt due to LAI, a model was constructed in which the absorptivity of the snow is multiplied with the incoming short-wave solar radiation obtained from local weather station data (http://178.217.169.232/sdss/index.php). Further factors considered included the number of days with temperatures ≥ 0 °C, the number of snowfall events, and the evolution of the snow covered area on the glaciers. To calculate the amount of snow melted based on the enthalpy of fusion of water (334 J/g), the snow pack was assumed to be at 0 °C. The mentioned factors and the snow albedo due to LAI were varied in 9 different scenarios (see SM Sec. 10 for details).

## Additional Information

**How to cite this article**: Schmale, J. *et al*. Modulation of snow reflectance and snowmelt from Central Asian glaciers by anthropogenic black carbon. *Sci. Rep.* **7**, 40501; doi: 10.1038/srep40501 (2017).

**Publisher's note:** Springer Nature remains neutral with regard to jurisdictional claims in published maps and institutional affiliations.

## Supplementary Material

Supplementary Information
