# Supplementary material for: Modulation of snow reflectance and snowmelt from Central Asian glaciers by anthropogenic black carbon
Source: Sci Rep. 2017 Jan 12;7:40501. doi: 10.1038/srep40501 (PMC5228185; doi:10.1038/srep40501)
Supplement: Supplementary Information [file srep40501-s1.pdf]

Supplementary Information

Modulation of snow reflectance and snowmelt from Central Asian glaciers by anthropogenic black carbon

Julia Schmale[1,2,*], Mark Flanner[3], Shichang Kang[4,5], Michael Sprenger[6], Qianggong Zhang[5,7], Junming Guo[7], Yang Li[7], Margit Schwikowski[2], Daniel Farinotti[8,9,10]

[1] Institute for Advanced Sustainability Studies, D-14467 Potsdam, Germany
[2] Paul Scherrer Institute, CH-5232 Villigen, Switzerland
[3] Department of Atmospheric, Oceanic and Space Sciences, University of Michigan, Ann Arbor, MI 48109-2143, USA
[4] State Key Laboratory of Cryospheric Sciences, Cold and Arid Regions Environmental and Engineering Research Institute, Chinese Academy of Sciences, 730000 Lanzhou, China
[5] Chinese Academy of Science Center for Excellence in Tibetan Plateau Earth Sciences, 100101 Beijing, China
[6] Institute for Atmospheric and Climate Science, ETH Zurich, CH-8092 Zurich, Switzerland
[7] Key Laboratory of Tibetan Environment Changes and Land Surface Processes, Institute of Tibetan Plateau Research, Chinese Academy of Sciences, 100101 Beijing, China
[8]Federal Institute of Forest, Snow and Landscape Research WSL, Birmensdorf, Switzerland
[9] GFZ German Research Centre for Geosciences, Section 5.4 - Hydrology, D-14473 Potsdam, Germany
[10] Laboratory of Hydraulics, Hydrology and Glaciology (VAW), Swiss Federal Institute of Technology, CH-8092, Switzerland

* corresponding author: julia.schmale@gmail.com

S 1 Sampling

Snow samples were taken from four different glaciers in Kyrgyzstan representing different mountain environments (Figure 1 of the main article and Table S1). Abramov glacier (39.61° N, 71.56° E) is located in the Pamir Alay Range (North-Western Pamirs) and covers an area of approximately 24 km^2 as of 2013 [1]. Glacier No. 354 (41.80° N, 78.15° E) and Suek Zapadniy (41.79° N, 77.75° E) are part of the Ak-Shiirak Range that belongs to the Inner Tien Shan and are located only 35 km apart. Glacier No. 354 extents roughly over 6.5 km^2 as of 2002 [2] and Suek covers about 1.0 km^2. Golubin glacier (42.46° N 74.49° E) is located 35 km south of the capital Bishkek in the Kyrgyz Range and extents over approximately 5 km^2. All glaciers are located at an altitude greater than 3600 m a.s.l. and samples were taken from elevations higher than 4000 m a.s.l. The glaciers experience precipitation year around based on local weather station data (`http://178.217.169.232/sdss/index.php`), and all had a negative mass balance during the last decade [1, 2, 3].

In terms of representativeness, Golubin is typical of the Western Tien Shan glaciers (Ala-Too Range) that are slightly lower-lying than the other glaciers and near urban agglomerations. Suek and Glacier No. 354 are representative for glaciers on the plateau south of the Issyk-Kl lake which are all very similar in terms of aspect, slope, elevation and form. The plateau is roughly 4000 m high, and on every north facing elevation (roughly 500 meters higher) small glaciers and on every south facing elevation small ice caps are located. Abramov is typical for the glaciers of the Northern Pamirs located in Kyrgyzstan with higher mountains in the surroundings.

Generally, each snow pit covered a full year of net snow accumulation from approximately August of the previous year to the sampling date, i.e. our samples represent aerosol deposition between August 2012 and August 2014. Snow pits were divided into equally deep layers representing one tenth of the total snow pit depth. In three cases (i.e. pits "No. 354 1 2013", "No. 354 2 2014", and "Suek 2 2014"; see Table S1 for nomenclature) snow pits were shallow and fewer samples were collected (see Table S1). In two cases no snow accumulation from the past 12 months was found (pits "No. 354 1 2014", and "Gol 2014") and samples were taken from open cracks that represent more than one year of snow accumulation. A total of 13 snow pits were dug and 226 snow samples taken. Layer depths ranged between 5 and 24 cm. Before taking samples, the first 5 cm of snow from the wall surface were scratched away with a clean stainless steel shovel to avoid contamination from other layers while digging the snow pit. 500 ml samples were taken following the "dirty hands, clean hands" protocol [4] with a stainless steel shovel and stored in Nalgene bottles. The bottles had been pre-cleaned by flushing them several times with ultra-pure water (Milli-Q $< 18.2\,\mathrm{m\Omega}$). After sampling the bottles were stored frozen in the dark until shipment to the laboratory. Upon arrival samples were processed immediately in a class 100 clean room. Additionally, snow density was determined in approximately 30 cm intervals. And, for each sampled layer the hand hardness index was taken, the grain size estimated and comments based on visual inspection were noted, e.g. ice lenses, dusty layers (see Table S3).

Table S1: Snow pit details. "Elev." is the elevation (m a.s.l.) at which the snow pit was dug. "Depth" (cm) is the total depth of the snow pit.

Name of pit	Coordinates	Elev.	Depth	Samples	Remarks
Abr 1 2013	39.59 °N 71.56 °E	4390	240	20	
Abr 2 2013	39.62 °N 71.52 °E	4275	125	20	
Abr 1a 2014	39.60 °N 71.56 °E	4381	168	20	Close to Abr 1 2013
Abr 1b 2014	39.60 °N 71.91 °E	4235	76	20	
Abr 2a 2014	39.62 °N 71.52 °E	4273	100	20	Close to Abr 2 2013
Abr 2b 2014	39.61 °N 71.53 °E	4200	71	20	
Suek 1 2013	41.78 °N 77.75 °E	4341	250	20	More than one year
Suek 1 2014	41.78 °N 77.75 °E	4325	116	20	Close to Suek 2013
Suek 2 2014	41.78 °N 77.75 °E	4473	39	8	Summit
No. 354 1 2013	41.80 °N 78.18 °E	4325	75	10	
No. 354 1 2014	41.80 °N 78.18 °E	4352	180	20	Close to No. 354 2013, several years
No. 354 2 2014	41.78 °N 78.17 °E	4355	52	8	
Gol 2014	42.44 °N 74.51 °E	4116	120	20	several years

S 2 Chemical analyses

S 2.1 Mineral Dust

The filters were pre-heated in an oxygen atmosphere at 800 °C for at least 2 hours. Samples were first sonicated for 15 minutes to avoid particle loss in the storage container bottles, then filtered twice and the filtration equipment was rinsed four times with ultrapure water ($< 18.2\,\mathrm{m}\Omega$) to avoid losses of particles [5]. Particle recovery rates are reported to be around 95 % [5, 6, 7, 8]. After filtration, the quartz fiber filters were stored in pre-cleaned glass weighing bottles and dried in glass vacuum desiccators.

S 2.2 Elemental analysis

Elemental concentrations were quantified using external calibration standards. An analytical standard was analyzed after the initial calibration after every 10 samples. Detection limits are given in Table S2. Repeated measurements of an externally certified reference solution (AccuTrace™Reference Standard) were performed to estimate the method's accuracy. Together with the recovery rates the accuracy ranged between 85 % for Cr and 105 % for Ni.

Table S2: Elements analyzed by ICP-MS and their detection limits.

Element	Detection limit
Al	0.084 ppb
Ti	0.103 ppb
V	0.015 ppb
Cr	0.029 ppb
Mn	0.009 ppb
Fe	1.235 ppb
Co	0.004 ppb
Ni	0.063 ppb
Cu	0.086 ppb
Zn	0.061 ppb
As	0.155 ppb
Cd	2.796 ppt
Pb	3.848 ppt
Bi	0.654 ppt

S 3 Impurity concentrations, composition of mineral dust

S 3.1 Impurity concentrations in snow

Table S4 shows the concentration profiles for EC, mineral dust and the Fe to dust ratio for each snow pit together with $\delta^{18}O$. The last column describes the snow state based on the code provided in Table S3. In some cases the snow pit depth corresponds to more than one year as indicated by (*) next to the snow pit name. These data were not included in the annual deposition flux calculations. Also, in some snow pits the $\delta^{18}O$ signature is difficult to interpret likely due to percolation processes. In such cases the annual accumulation was approximated through the visibly and moderately dirty snow layers that are indicative of summer dust deposition on all glaciers.

Table S3: Snow state description code used in Table S4.

Code	Snow state description
1	moderately dirty, a bit icy, coarse grains, melt forms
2	fresh snow, very fine grain
3	visibly dirty, few days old snow, fine grain
4	moderately dirty, few days old snow, fine grain
5	clean, few days old snow, fine grain
6	ice lens
7	mixed grain snow

Table S4: Snow pit data for Abramov glacier. Depth in cm and mm water equivalent (w.e.), isotopic composition, EC and dust concentrations, as well as Fe/dust ratio. The key to "Code", characterizing the snow state description, is given in Table S3. (*) indicates that the snow pit represents >1 year.

Depth (cm)	Depth (mm w.e.)	$\delta^{18}O/\delta^{16}O$ (‰)	EC (ng/g w.e.)	Dust (μg/g w.e.)	Fe/dust (g/g)	Code
**Abramov 1a 2013**						
-24	-121.01	-11.75	242.96	41.87	0.008	3
-48	-242.02	-13.68	23.76	4.37	0.008	3
-72	-366.81	-18.17	14.24	2.25	0.006	5
-96	-489.07	-24.28	7.25	N.A.	N.A.	5
-120	-611.34	-28.10	14.13	1.68	0.007	5
-144	-749.82	-12.97	8.00	0.39	0.012	5
-168	-874.79	-15.45	8.19	2.58	0.003	5
-192	-902.91	-11.58	11.68	1.11	0.010	5
-216	-996.82	-8.24	29.19	7.12	0.003	5
-240	-1015.77	-7.65	429.95	154.19	0.004	3
**Abramov 2a 2013**						
-12.5	-63.49	N.A.	177.93	82.31	N.A.	3
-25.0	-126.97	-11.44	122.97	24.42	0.024	3
-37.5	-190.46	-12.55	50.26	6.67	0.017	5
-50.0	-253.94	-13.77	31.68	7.11	0.007	5
-62.5	-317.43	-14.14	31.16	6.52	0.004	5
-75.0	-380.91	-15.03	19.94	5.08	0.006	5
-87.5	-444.40	-14.60	14.50	6.43	0.003	5
-100.0	-507.89	-13.01	38.23	8.35	0.002	5
-112.5	-524.95	-11.49	70.46	14.80	0.003	5
-125.0	-571.37	-12.23	353.23	82.74	0.001	3

Continued on next page.

Table S4: Continued from previous page (snow pit data for Abramov glacier).

Depth (cm)	Depth (mm w.e.)	$\delta^{18}O/\delta^{16}O$ (‰)	EC (ng/g w.e.)	Dust (μg/g w.e.)	Fe/dust (g/g)	Code
**Abramov 1a 2014**						
-19	-107.22	-8.96	95.28	2.20	0.017	2
-32	-155.88	-13.90	354.66	40.25	0.013	3
-48	-233.82	-15.45	219.71	39.22	0.008	1
-65	-316.63	-16.10	114.91	16.11	0.004	6
-81	-455.33	-16.34	51.41	6.29	0.012	5
-97	-495.19	-15.17	23.68	1.03	0.019	5
-113	-530.43	-17.17	10.18	1.38	0.004	5
-129	-605.54	-16.66	17.81	3.23	0.006	5
-145	-680.64	-15.82	90.32	16.67	0.008	5
-168	-1092.89	-13.32	1098.31	156.25	0.008	1
**Abramov 1b 2014**						
-15	-28.25	-12.96	55.05	7.96	0.005	2
-22	-41.44	-13.44	183.66	38.46	0.001	2
-29	-152.94	-14.02	586.83	64.15	0.006	3
-36	-189.86	-14.34	506.38	39.47	0.010	5
-43	-210.57	-14.12	291.77	20.51	0.013	5
-50	-244.85	-14.50	195.63	18.40	0.012	5
-57	-279.13	-14.40	340.74	37.44	0.010	5
-64	-361.63	-12.04	94.44	11.66	0.007	1
-71	-401.18	-9.95	232.48	37.62	0.007	1
-76	-429.44	-13.66	2114.53	223.08	0.008	6
**Abramov 2a 2014**						
-10	-43.57	-13.55	502.04	69.61	0.013	1
-20	-87.14	-13.25	237.69	33.52	0.010	2
-30	-130.71	-14.23	102.03	8.25	0.009	3
-40	-177.94	-14.92	77.74	6.96	0.010	4
-50	-222.42	-15.75	70.18	7.02	0.017	5
-60	-266.91	-15.73	133.91	15.35	0.009	5
-70	-311.39	-13.49	551.37	40.10	0.009	5
-80	-355.88	-14.25	937.29	70.00	0.009	5
-90	-400.36	-11.33	239.22	43.37	0.010	3
-100	-912.87	-8.71	41.70	7.27	0.013	1
**Abramov 2b 2014**						
-8	-45.20	-12.61	747.61	167.57	0.011	1
-15	-84.76	-12.26	1013.95	138.75	0.010	1
-22	-107.74	-11.93	468.38	49.78	0.010	1
-29	-142.02	-13.18	192.26	20.25	0.012	5
-36	-176.29	-13.30	83.43	9.43	0.013	5
-43	-226.77	-13.08	112.28	11.74	0.012	5
-50	-263.69	-13.67	205.37	28.40	0.010	5
-57	-300.61	-12.76	160.76	25.68	0.011	5
-64	-289.30	-12.73	89.12	7.65	0.012	5
-71	-320.95	-11.22	609.91	122.47	0.009	5

Table S5: Snow pit data for Suek glacier and glacier No. 354. Depth in cm and mm water equivalent (w.e.), isotopic composition, EC and dust concentrations, as well as Fe/dust ratio. The key to "Code", characterizing the snow state description, is given in Table S3. (*) indicates that the snow pit represents >1 year.

Depth (cm)	Depth (mm w.e.)	$\delta^{18}O/\delta^{16}O$ (‰)	EC (ng/g w.e.)	Dust (μg/g w.e.)	Fe/dust (g/g)	Code
**Suek 1 2013 (*)**						
-24	-84.65	-11.09	14.90	N.A.	N.A.	2
-46	-162.24	-12.20	601.64	334.10	N.A.	7
-68	-288.61	-12.94	39.95	5.81	N.A.	5
-90	-381.98	-12.40	340.93	142.15	0.006	7
-112	-475.35	-10.64	53.73	10.70	0.011	5
-134	-607.40	-10.91	242.70	122.28	0.014	5
-156	-707.12	-11.06	26.81	5.76	0.013	6
-178	-844.05	-11.98	54.69	15.05	0.006	5
-200	-948.37	-14.04	90.25	43.64	0.006	6
-250	-1133.21	-12.33	92.70	54.03	0.006	6
**Suek 1 2014**						
-10	-42.87	-13.80	915.74	212.77	0.007	1
-20	-85.75	-10.92	1621.36	131.25	0.015	1
-30	-128.62	-10.17	279.60	42.00	0.021	1
-40	-189.89	-9.71	640.89	177.50	0.009	4
-50	-237.36	-9.51	504.05	111.11	0.013	3
-66	-254.07	-9.72	249.58	59.72	0.029	6
-76	-292.57	-9.75	175.12	29.63	0.015	6
-86	-329.61	-9.83	126.45	14.23	0.011	5
-96	-361.70	-9.74	146.44	29.03	0.013	5
-116	-367.94	-9.96	347.42	98.82	0.012	6
**Suek 2 2014**						
-7	-21.10	-12.75	103.57	12.71	0.001	5
-17	-51.23	-14.56	12.15	2.19	0.026	5
-24	-72.33	-12.44	104.46	12.50	0.345	5
-39	-117.53	-8.29	3446.68	288.89	0.000	1
**No. 354 1 2013**						
-17	-53.64	-9.85	52.73	27.39	0.002	2
-31.5	-139.30	-11.25	518.69	302.63	0.011	4
-46	-203.43	-12.79	130.45	19.75	0.014	5
-60.5	-267.55	-12.41	70.17	62.92	0.002	5
-75	-331.67	-9.79	221.37	121.37	0.006	4
**No. 354 1 2014 (*)**						
-20	-100.45	-14.77	2419.03	436.51	0.012	1
-40	-200.91	-13.56	292.26	57.04	0.009	3
-60	-301.36	-12.87	1599.65	369.39	0.014	7
-80	-401.81	-13.35	206.58	34.91	0.017	7
-100	-502.26	-13.17	121.57	19.81	0.015	7
-120	-602.72	-12.92	193.00	30.43	0.038	5
-140	-703.17	-12.85	222.46	42.34	0.010	6
-160	-803.62	-12.31	258.12	65.77	0.013	3
-180	-904.08	-11.98	917.81	81.82	0.012	7
**No. 354 2 2014**						
-9	-33.90	-10.30	67.26	4.91	0.007	2
-22	-116.02	-10.67	1617.09	335.09	0.012	2
-32	-160.72	-11.27	661.62	79.00	0.014	3
-42	-210.95	-14.35	584.95	128.26	0.015	3

Table S6: Snow pit data for Golubin glacier. Depth in cm and mm water equivalent (w.e.), isotopic composition, EC and dust concentrations, as well as Fe/dust ratio. The key to "Code", characterizing the snow state description, is given in Table S3. (*) indicates that the snow pit represents >1 year.

Depth (cm)	Depth (mm w.e.)	$\delta^{18}O/\delta^{16}O$ (‰)	EC (ng/g w.e.)	Dust (μg/g w.e.)	Fe/dust (g/g)	Code
**Golubin 2014 (*)**						
-12	-76.80	-10.44	963.76	111.29	0.004	1
-24	-153.60	-10.54	1342.44	155.22	0.010	7
-36	-230.40	-10.28	574.00	51.40	0.011	6
-48	-307.20	-10.28	455.25	40.00	0.011	7
-60	-384.00	-10.67	512.28	45.95	0.012	7
-72	-460.80	-10.90	443.82	37.27	0.011	6
-84	-537.60	-11.32	258.28	25.95	0.014	7
-96	-614.40	-11.35	206.20	19.92	0.018	7
-108	-691.20	-10.94	576.81	97.67	0.009	7
-120	-768.00	-11.78	1972.92	102.00	0.010	6

S 3.2 Elemental composition of dust

Table S7 compares ratios of various elements with Al from dust in snow samples in the Tien Shan and Northern Pamirs with soil dust in of the Aral Sea [9] basin. The large differences between the Aral Sea and snow dust samples indicate that the Aral Sea is most likely not an important contributor to dust deposition in the sampled mountain ranges. Elemental concentration were determined in both cases with ICP-MS. Sample digestions methods, however, differ. In ref. [9] a full digestion was performed which leads to higher extraction efficiencies [10] for the various elements. Taking this into account differences in the ratios are still large enough to support the conclusion.

Table S7: Ratios of ions and elements with Al for mineral dust in our samples (Tien Shan, Northern Pamirs) and for the Aral Sea [9].

Location	Cr	Mn	Ni	V	Zn	Co	Cu	Ti	As	Ba	Pb
Tien Shan	0.00	0.06	0.00	0.00	0.02	0.00	0.00	0.03	0.00	19.04	25.96
Northern Pamirs	0.00	0.07	0.00	0.00	0.06	0.00	0.01	0.03	0.00	23.63	11.29
Aral Sea	83	217	20	33	24	4	7	600	5	133	6

S 4 Back trajectory and footprint analysis

Figure S1 gives a visual impression of the derived footprints for Suek Zapadniy (also representative for No. 354) and Golubin for DJF and JJA 2013. Data for Abramov is shown in Fig. 1 of the main article.

Figures S2–S4 show the anthropogenic and natural fire BC emission footprints for the same time periods and glaciers, while Figure S5 provides a summary on the air mass origin per region based on the footprint calculations for each glacier as averages over all seasons between August 2012 and August 2014.

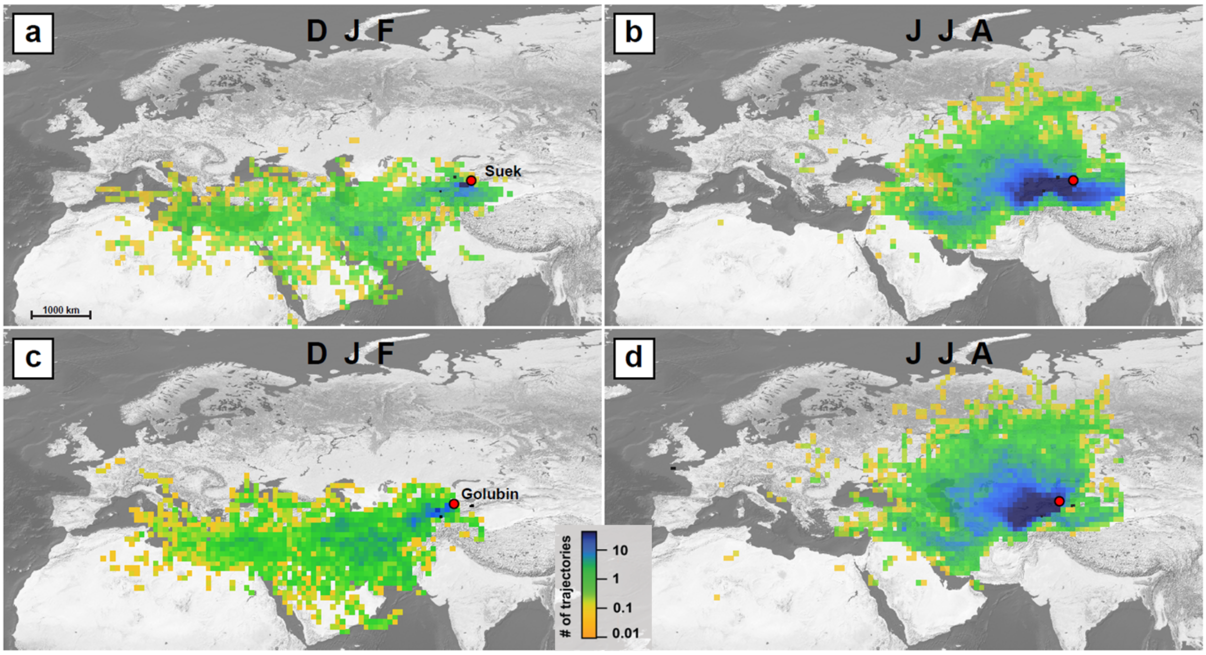

Figure S1: Footprints of air mass back trajectories launched over Suek Zapadnyi (a,b) and Golubin (c,d) in winter (DJF) and summer (JJA). The background maps were created with Quantum GIS v.2.6 (http://www.qgis.org/de/site/) using Natural Earth I raster maps (http://www.naturalearthdata.com/downloads/10m-raster-data/10m-). The emissions and back trajectory footprints were created with Igor Pro v.6 (https://www.wavemetrics.com/) and overlaid.

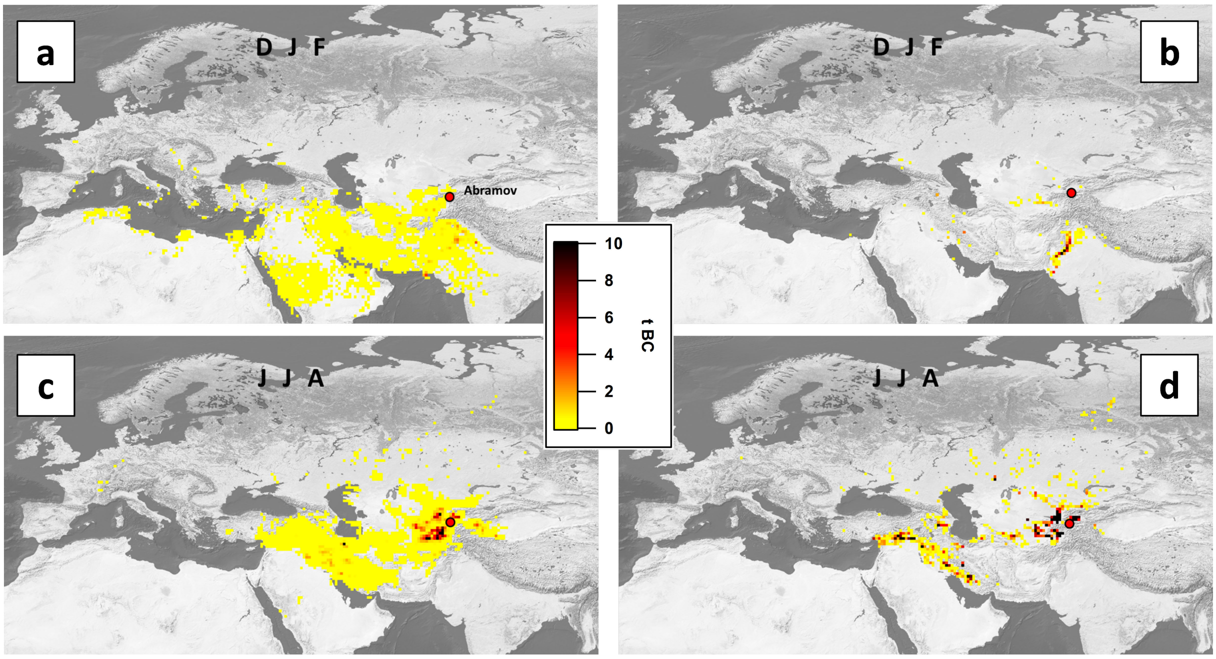

Figure S2: Footprints for anthropogenic (a,c) and natural fire (b,d) BC emissions in winter (top) and summer (bottom) for Abramov. Fire emissions are from 2013 while anthropogenic emissions are from 2010. The color code indicates the total mass of BC emitted per season. For fire BC emissions values have to be multiplied by 10^{-2}. The background maps were created with Quantum GIS v.2.6 (http://www.qgis.org/de/site/) using Natural Earth I raster maps (http://www.naturalearthdata.com/downloads/10m-raster-data/10m-). The emissions and back trajectory footprints were created with Igor Pro v.6 (https://www.wavemetrics.com/) and overlaid.

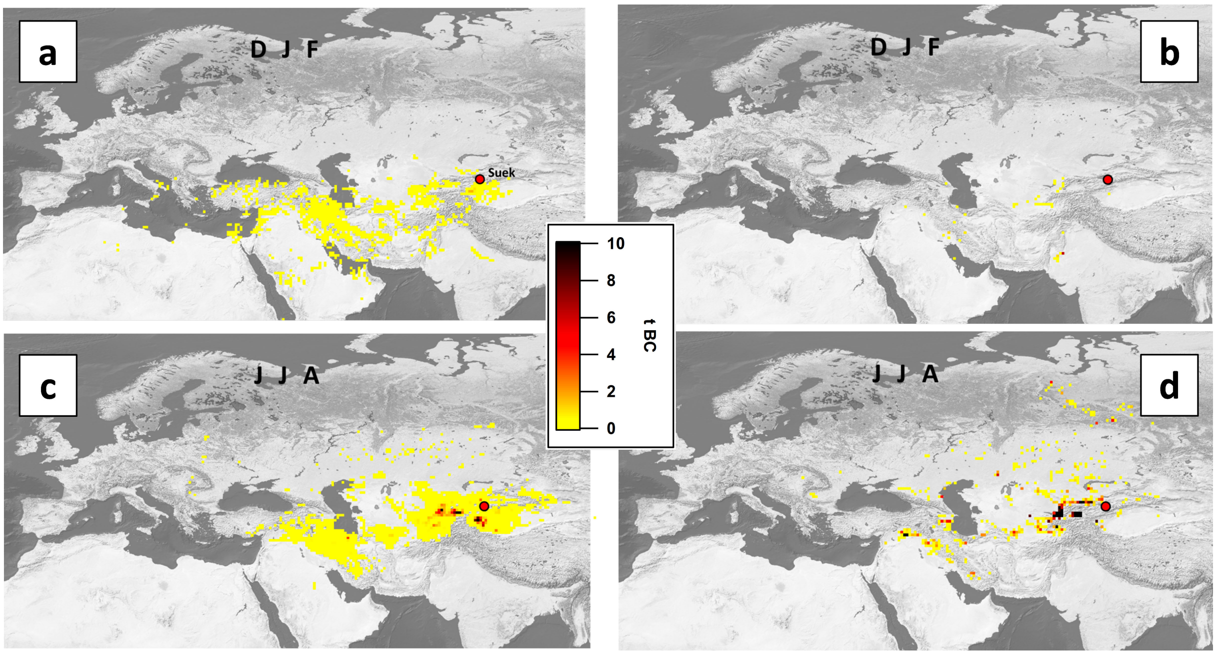

Figure S3: Same as Figure S2 but for Suek Zapadniy. The background maps were created with Quantum GIS v.2.6 (http://www.qgis.org/de/site/) using Natural Earth I raster maps (http://www.naturalearthdata.com/downloads/10m-raster-data/10m-). The emissions and back trajectory footprints were created with Igor Pro v.6 (https://www.wavemetrics.com/) and overlaid.

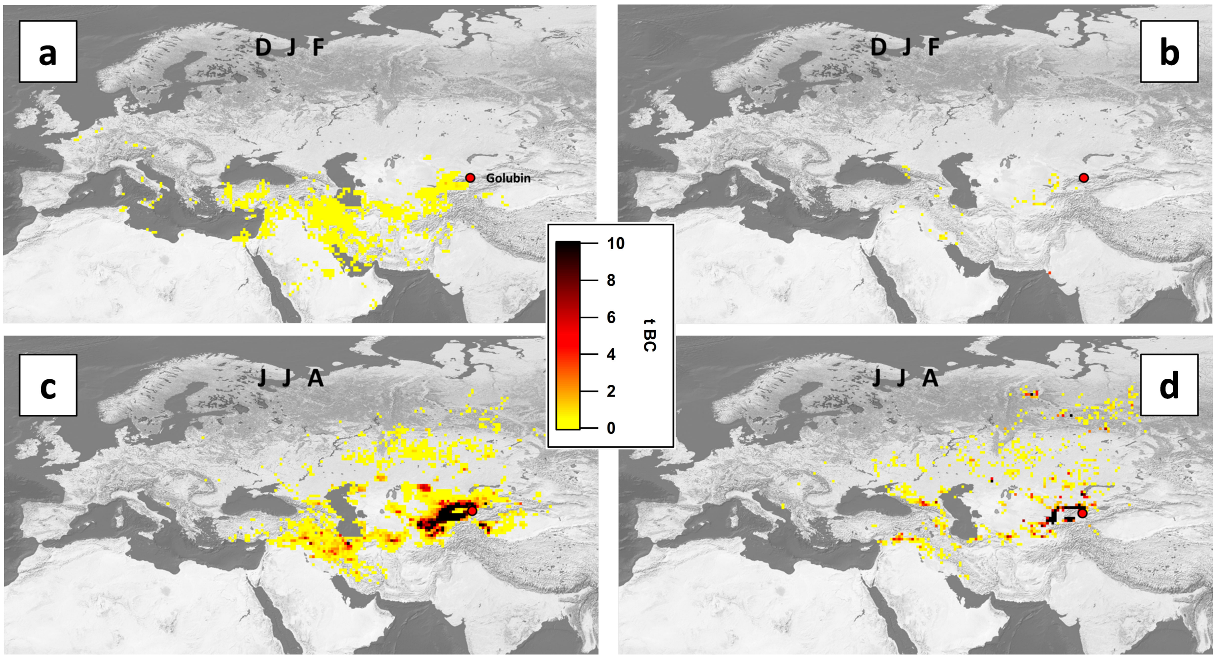

Figure S4: Same as Figure S2 but for Golubin. The background maps were created with Quantum GIS v.2.6 (http://www.qgis.org/de/site/) using Natural Earth I raster maps (http://www.naturalearthdata.com/downloads/10m-raster-data/10m-). The emissions and back trajectory footprints were created with Igor Pro v.6 (https://www.wavemetrics.com/) and overlaid.

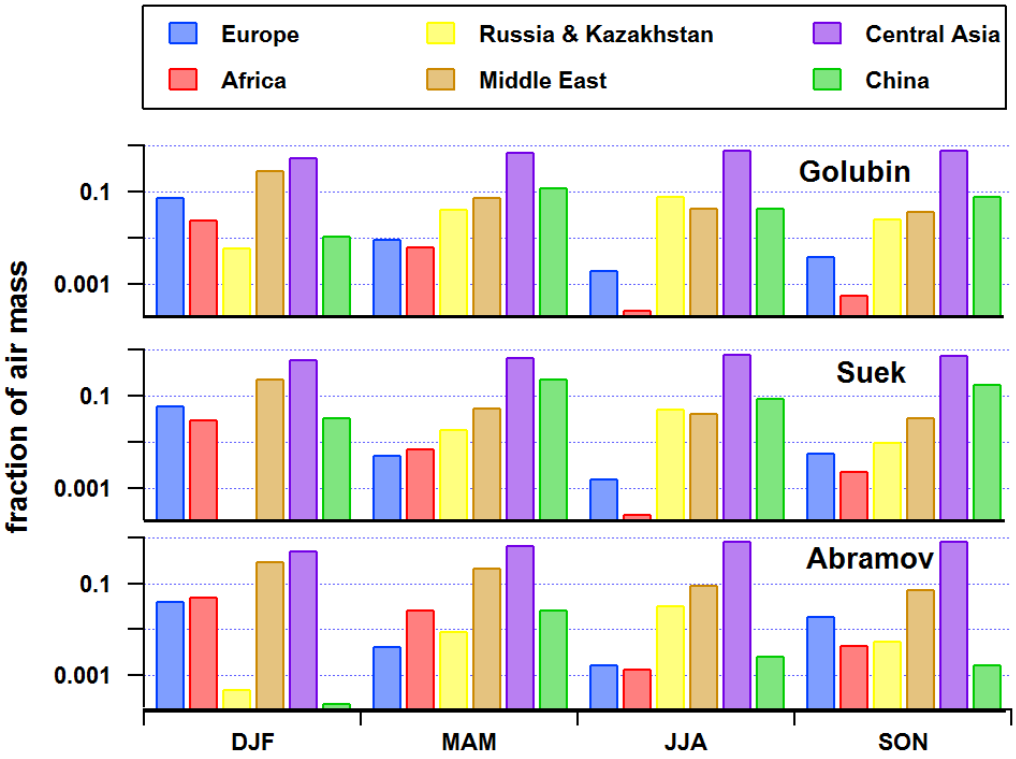

Figure S5: Air mass origin per region from the footprint analysis of Suek apadniy (top), Golubin (middle), and Abramov (bottom).

S 5 Contribution of anthropogenic and natural fire BC emissions

Figure S6 shows the theoretical contribution of BC in tons per season to the release spot of the back trajectories on each glacier and the fraction of BC from natural fires. The mass is derived from the calculated residence time of the back trajectories in the boundary layer per season multiplied by the emission flux in 2010 from the Eclipse V5 inventory for the anthropogenic contribution. The same calculation is performed for the Finn v1.5 natural fire emissions flux in 2012 - 2014. Note that this analysis is not quantitative and does not take into account wet and dry deposition. Also, we assume that the anthropogenic emissions have not changed significantly from 2010 compared to the period from 2012 to 2014 so that the comparison of natural and anthropogenic BC contribution is still reasonable. The relative difference between the BC contributions provides information on regional and seasonal differences.
Figure S7 shows the same information but provides details on the regional contribution to anthropogenic and natural fire BC.

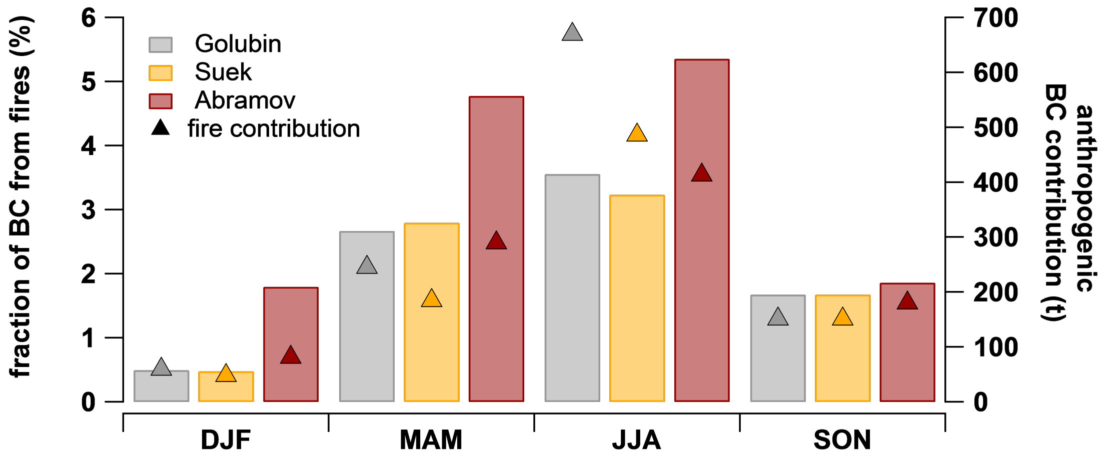

Figure S6: Theoretical contribution of anthropogenic BC to different glaciers per season. The triangles represent the fraction of BC from natural fires.

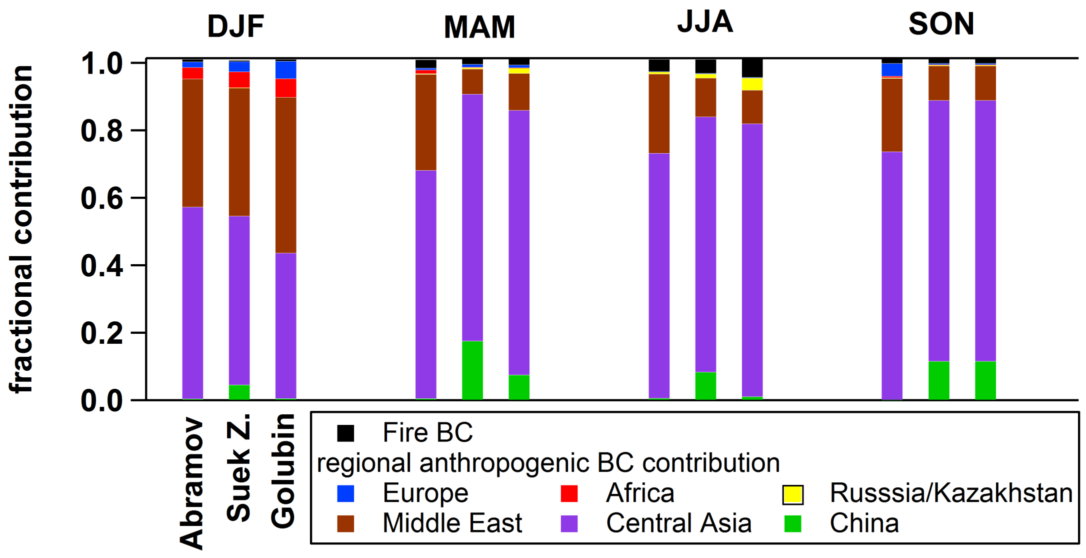

Figure S7: Fractional contribution of regional anthropogenic BC (colors) emissions and total natural fire BC (black) contribution for Abramov, Suek Zapdniy and Golubin for each season.

S 6 Comparison with a global model

In ref. [11] the Community Atmosphere Model (CAM) version 5.2 was run with the ECLIPSEv4a data set [12] for anthropogenic emissions and GFEDv3 [13] for natural fire emissions. Table S8 shows annual average results from the CAM calculations, which include explicit wet and dry deposition processes, comparing them to our back trajectory approach. The regional subdivision for the CAM study included the USA, Canada, Nordic countries, Russia, other Europe, Asia and the rest of the world. Here we compare data from the Nordic countries and other Europe to our Europe domain, Russia with Russia / Kazakhstan and the total anthropogenic and natural fire contributions. The CAM model results provide additionally information on the anthropogenic BC source type contribution from the overall domain.

Table S8: Anthropogenic BC contribution at three glaciers by region as calculated by CAM5.2 (Eclipse V4a, GFED v3) and this study's (Eclipse V5, Finn v1.5) back trajectory approach.

Glacier	Region	Source type	BC contribution (%)	
			CAM5.2	This study
Abramov	Total		94.6	97.9
	Europe		2.6	1.2
	Russia / Kazakhstan		1.9	1.3
		Energy+Industry+Waste	10.9	
		Domestic	41.7	
		Transport	24.7	
		Agricultural Waste Burning	5.8	
		Flaring	11.6	
		Grass / Forest Fires	5.4	2.1
Suek	Total		94.5	98.3
	Europe		4.1	1.1
	Russia / Kazakhstan		6.2	0.5
		Energy+Industry+Waste	12.9	
		Domestic	38.8	
		Transport	24.9	
		Agricultural Waste Burning	8.0	
		Flaring	9.8	
		Grass / Forest Fires	5.6	1.7
Golubin	Total		94.6	97.6
	Europe		4.2	1.7
	Russia / Kazakhstan		5.8	1.4
		Energy+Industry+Waste	14.5	
		Domestic	36.0	
		Transport	24.6	
		Agricultural Waste Burning	8.8	
		Flaring	10.6	
		Grass / Forest Fires	5.4	2.4

S 7 Heavy Metal Enrichment

Figure S8 shows the enrichment factors of heavy metals relative to the upper continental crust. Values > 10 indicate anthropogenic contamination. Similar factors have been found on Fedchenko glacier, Central Pamirs [14]. Note that heavy metals can also originate from natural sources to a certain degree, especially in arid regions [15]. However, the very high enrichment factors (e.g., ca. 100 for As and Cd) point towards regional anthropogenic sources. Typical sources are non-ferrous metal production, as well as recovery and refining of natural gas and oil – activities found across the former Soviet Union countries, China, and Iran. These potential source regions are coherent with the results from the back trajectory analysis.

Even though the here considered heavy metals are not necessarily emitted by the same sources as anthropogenic BC, they most likely arrive with the same air masses that have to be lifted from the boundary layer to the elevation of the glaciers. Heavy metals and BC hence most likely present similar emission regions so that heavy metals are indicative of anthropogenic BC sources.

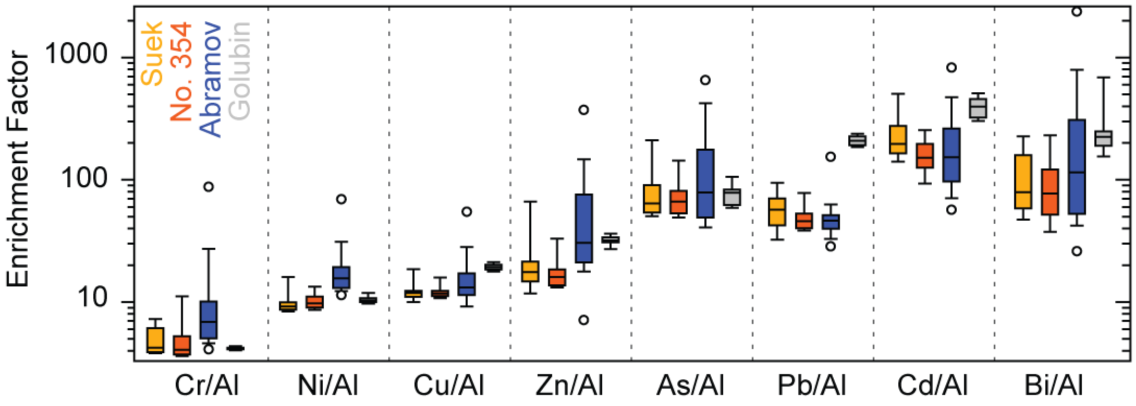

Figure S8: Heavy metal enrichment factors relative to the upper continental crust.

S 8 SNICAR Model

We applied a single-column, multi-layer version of the SNICAR model with 10 nm spectral resolution between wavelengths of 0.3 and 5.0 μm. A single layer version of this model can be operated online at http://snow.engin.umich.edu. Snow grain sizes were derived from the stratigraphy, ranging from 100 μm for fresh, clean snow to 1500 μm for old, icy and dirty snow, and were varied in low, central and high grain size scenarios in the model runs. Specifically, we applied snow grain sizes listed in Table S9 based on the layer-specific snow types identified in the stratigraphy.

The absorptivity of the dust particles was approximated by the Fe content of the mineral dust (mass ratio of mineral dust to Fe in uppermost snow layer = 0.0083, $R = 0.85$). Based on ref. [16], 55% of the Fe was assumed to reside in absorptive iron oxides, and the ratio of hematite to goethite was assumed to be 0.7 in this region [17]. Further we multiply the Fe mass fraction with 1.43 for hematite and 1.59 for goethite to account for their molecular mass and divide by the minerals' densities of 5.28 and 4.3, respectively, to derive their volume fractions.

A dust particle size distribution of 1.85 μm geometric mean diameter by volume and a geometric standard deviation of $\sigma_{\rm g} = 2.0$ was applied based on ref 18 which is similar to our measurements (see Figure S9).

The model was run at 30-minute time-steps for the days on which each snow pit was dug, for both clear sky and all sky conditions. All sky forcing was derived from local down-welling short-wave radiation measurements collected near each glacier from the Central Asian Water project (CAWa, `http://178.217.169.232/sdss/index.php`).

To estimate further uncertainties in the albedo reduction in addition to the snow grain size scenarios, we repeat the calculations with the twofold (halved) LAI concentrations. The extreme low scenario reflects the case with small grain sizes and half of the impurity concentrations, while the extreme case stands for large grain sizes and the doubled impurity concentration. It has to be noted that a factor 2 difference for dust concentration is beyond the expected analyses uncertainties. Also for EC, assuming half of the concentration allows for a margin that is most likely beyond the expected uncertainty. As discussed in the methods section, EC concentrations are more likely under than over-estimated already.

Table S9: Snow grain sizes (μm) used for the albedo calculation with the SNICAR model.

Snow state	Scenario		
	central	low	high
Fresh snow, very fine grain	100	50	200
Clean, few days old snow, fine grain	250	125	500
Moderately dirty, few days old snow, fine grain	250	125	500
Visibly dirty, few days old snow, fine grain	250	125	500
Mixed grain snow	250	125	500
Moderately dirty and icy, coarse grains	1000	500	2000
Ice lens	1500	750	3000

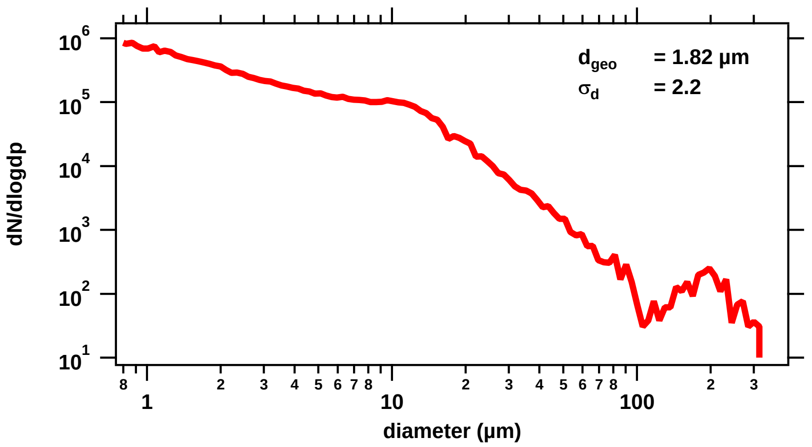

Figure S9: Median dust particle size distribution of all surface layer samples.

S 9 Snow type classification

We classify all snow pits according to surface snow types typical for summer conditions in the region. Fresh snow is classified as snow younger than one day when we witnessed the snow fall. Fresh snow contains typically relatively low LAI concentrations. Several days old snow is defined as snow older than one day without melt forms. Melt forms are loosely defined as snow crystals that visibly experienced melting and constitute the third type of encountered surface snow. EC concentrations in several days old snow are 3.1 ± 2.0 times higher than in fresh snow. Concentrations are higher likely due to dry deposition of LAI while the snow surface is exposed for several days as has been found by other studies [18, 19]. For mineral dust, there is an average enrichment by a factor 4.3 which, however, is statistically not significant. The higher variability in the mineral dust concentrations opposed to EC could be due to different emission mechanisms. While EC is released more regularly through continuous human activity, mineral dust needs to be lifted by wind that is strong enough and which does not necessarily occur regularly. These surface layers are often the second dirtiest in their snow pits (see Table S10). Concentrations increase again in snow with melt forms compared to several days old snow by a factor 6.3 ± 5.0 for EC and 4.4 ± 4.6 for mineral dust. This further increase occurs because these LAI are insoluble and can hence not percolate through the snow but are retained at the surface [20]. Surface layers of this type are often the dirtiest or second dirtiest layers in the snow pits. Typically, the dirtiest layers are found at the very bottom of the snow pit where they represent the surface snow layer with melt forms from the previous summer, or at the surface representing the current summer. In some cases, the dirtiest layers are also encountered in the second layer, mostly due to a fresh snow cover, but also occasionally under top layers with melt forms. In both cases the dirtiest layers also represent the current summer deposition.

The average surface albedo reduction and RF grouped by snow type hence represent the typical surface snow conditions on Tien Shan and Northern Pamir glacier accumulation zones that are representative for summer conditions. An extrapolation across the glaciated area of Kyrgyzstan is hence reasonable, as most glacier accumulation zones are located at similar elevations (lower is not possible due to the climatic conditions, and there are only few higher mountain areas). Also, comparable concentrations of LAI can be expected as we use data representing highly exposed (Golubin), slightly remote (Suek Zapadniy, No. 354) and remote glaciers (Abramov).

Table S10: Classification of snow pits according to their surface snow type. Concentration of EC (ng g^{-1}) and mineral dust ($\mu g\ g^{-1}$) are given for both, the surface layer and the dirtiest layer in the snow pit. "Rank" indicates how many layers were dirtier than the surface snow layer out of the total number of layers. E.g., 9/10 means that 9 layers out of 10 were dirtier. "Layer" indicates the layer, counted from the surface, for wich the highest EC and mineral dust concentration is found. E.g., 10-2 means that the highest EC and dust concentrations were both found in layer 10 and 2, respectively.

Snow pit	Snow type	Surface layer concentration				Highest concentration in snow pit		
		EC	Rank	Dust	Rank	EC	Dust	Layer
No. 354 2 2014	fresh	67.26	9/10	4.91	9/10	1617.09	335.09	2-2
No. 354 1 2013	fresh	52.73	4/5	27.39	3/5	518.69	302.63	2-2
Abr 1a 2014	fresh	95.28	4/10	2.20	8/10	1098.31	156.25	10-10
Suek 1 2013	fresh	14.90	9/10	N.A.	9/10	601.64	334.10	2-2
Abr 1b 2014	fresh	55.05	9/10	7.96	9/10	2114.53	223.08	10-10
Suek 2 2014	several days old	103.57	2/4	12.71	1/4	3446.68	288.89	4-4
Abr 1a 2013	several days old	242.96	1/10	41.87	1/10	429.95	154.19	10-10
Abr 2a 2013	several days old	177.93	1/10	82.31	1/10	353.23	82.74	10-10
No. 354 1 2014	melt	2419.03	0/10	436.51	0/10	2419.03	436.51	1-1
Gol 2014	melt	963.76	2/10	111.29	1/10	1972.92	155.22	10-2
Abr 2a 2014	melt	502.04	2/10	69.61	1/10	937.29	70.00	7-7
Abr 2b 2014	melt	747.61	1/10	167.57	0/10	747.61	167.57	1-1
Suek 1 2014	melt	915.74	1/10	212.77	0/10	1621.36	212.77	2-1

S 10 Snow melt estimation

To estimate the snow melt due to LAI, a model was constructed in which the absorptivity of the snow is multiplied with the daily average incoming short-wave solar radiation obtained from local weather station data (`http://178.217.169.232/sdss/index.php`). The average albedo for each snow type as derived with the SNICAR model (Fig. 3a in the main manuscript) was used to calculate melt induced by (1) melt of fresh snow without impurities, (2) melt of snow with dust and BC impurities, and (3) melt of snow with only anthropogenic BC contamination (Table S11). The effect of LAI is calculated by comparing the result from (1) with (2) or (3). At each snowfall event, and for each day without snowfall (and an average temperature $> 0°C$), the albedo for "fresh snow" and "several days old snow" ("snow with melt forms") was assumed, respectively. The numerical values for the assumed albedo are given in Table S11.

Factors derived from local weather station data included (1) the number of days per month with average temperature $\geq 0°C$, as a proxy for the number of days with snow melt, (2) the number of days per month with snowfall (complete snow cover was assumed within 0.5 and 1 days depending on the air temperature), and (3) the daily solar shortwave insolation averaged per month (used to calculate the energy flux at the surface).

The evolution of the snow cover on the glaciers was taken from Fig. 4 in ref. [1] in the case of Abramov. For the other glaciers our own observations from July and August 2013 and 2014 and observations by local glaciologists were used. To calculate the amount of snow melted based on the enthalpy of fusion of water (334 $J\,g^{-1}$), the snow pack was assumed to be at 0°C.

The basic model equations are:

$$M_{fresh} = N_{\ snowfall} \cdot N_{T>0,\,f} \cdot \alpha_1 \cdot SW \tag{1}$$

$$M_{aged} = N_{T>0} \cdot \alpha_2 \cdot SW \cdot SCAF \tag{2}$$

$$M_{meltform} = N_{T>0,\,5d} \cdot \alpha_3 \cdot SW \cdot SCAF \tag{3}$$

$$M_{total} = M_{fresh} + M_{aged} + M_{meltform} \tag{4}$$

with:

$N_{snowfall}$ = number of days with snowfall

$N_{T>0,\,f}$ = number of days with temperature > 0 when fresh snowfall occured

$N_{T>0}$ =number of days with temperature> 0 for a consecutive period < 5 days

$N_{T>0,\,5d}$ = number of days with temperature> 0 occurring after a consecutive period of 5 days with temperature> 0

α_1 = albedo of fresh snow

α_2 = albedo of aged snow

α_3 = albedo of snow with melt forms

SCAF = average snow covered area fraction

SW = shortwave radiation

To estimate the overall uncertainty of the melt rate we include the following variabilities:

(a) range of albedo values from the SNICAR model calculations (see SM Sec. 8 and Fig. 3 (a))

(b) sublimation and snow temperature < 0 C

(c) variability in the meteorogolical conditions.

With respect to (a), we apply the central estimate and the widest range of albedo values for each snow type.

For (b) we calculate melt for snow at $0C$ and at $-5C$ to account for the potential energy that is needed to warm the snow before it can melt. We also consider the possibility that snow

sublimates. Sublimation occurs when the latent heat flux is negative. We derive the latent heat flux based on equation 5 in ref. [21]:

$$H_{la} = 0.622 \cdot rho_{air} \cdot L_v \cdot C_{se} \cdot v \cdot (e_a - e_s)/P \tag{5}$$

with:
H_{la} = latent heat flux
rho_{air} = density of air at ambient pressure and temperature
L_v = latent heat of vaporization (2600000 J kg^{-1})
C_{se} = bulk turbulent exchange coefficient (0.00153), taken from ref. [21]
e_a = water vapor pressure, derived from ambient relative humidity measurement data and the saturation water vapor pressure calculated with the Tetens formula
e_s = vapor pressure at the surface (611 Pa)
P = ambient pressure

All variables were taken from the nearest automated weather stations.
The average latent heat flux for JJA on Abramov is -19 W m^{-2}, on Suek -25 W m^{-2}, and -3 W m^{-2} on Golubin.

For (c) different cases were calculated including variations of ±25% in (a) the estimated snow cover fraction, (b) the number of days with melting, and (c) the number of days with snow fall. Results for each case are expressed as minimum (−25%), maximum (+25%) and central estimate.
From the combination of all variables we show the range of results for the meteorological variability including sublimation and snow warming (Fig. 3 c-e), coloured bars) and add as extreme case results for twofold (halved) LAI concentrations.
The results are compared to calculations derived from the model ensamble of ref. [3]. In those models, which were calibrated to direct measurements of both ablation and accumulation, monthly melt rates from the 1960s to 2012 were calculated based on meteorological reanalysis data. Data between 2004 and 2012 from the models was used to calculate the median and interquartile range of the snow melt in JJA, for which our albedo data is most representative.

Table S11: Albedo for different snow types according to snow melt case averaged over all glaciers. The values in parenthesis reflect albedo when LAI concentrations were doubled or halved.

Case	grain size	fresh	several days old	with melt forms
clean	central	0.80	0.76	0.76
BC and dust	central	0.76	0.70	0.65
anthropogenic BC	central	0.78	0.71	0.68
clean	low	0.82	0.80	0.80
BC and dust	low (half)	0.78 (0.79)	0.75 (0.76)	0.70 (0.72)
anthropogenic BC	low (half)	0.80 (0.81)	0.76 (0.77)	0.72 (0.74)
clean	high	0.76	0.74	0.74
BC and dust	high (double)	0.73 (0.72)	0.64 (0.62)	0.56 (0.54)
anthropogenic BC	high (double)	0.74 (0.73)	0.65 (0.63)	0.57 (0.55)

S 11 Fe Sensitivity Study

We are likely to underestimate the Fe concentration in mineral dust by a factor 1.5 to 5 [10, 22, 23, 24] due to the method of sample digestions (see Methods). Given that we find an Fe fraction of roughly 1% and that the fraction in the upper continental crust is about 3% [25], a factor 3 seems plausible. For that reason we performed the albedo change and snowmelt calculations again assuming a 3% Fe mass fraction in mineral dust.

The albedo of snow type fresh with mineral dust and BC contamination would remain the same (0.76) due to the low impurity concentration, while for several days old snow (snow with melt forms) it would decrease from 0.70 to 0.68 (0.65 to 0.62). In terms of order of importance for snowmelt this means that in several days old snow BC would only be 1.03 times more important than mineral dust opposed to 1.29 assuming a 1% Fe mass concentration. For snow with melt forms, BC remains clearly more important with a factor of 1.26 versus 1.71. The actual induced snowmelt is rather insensitive to the change in mineral dust absorptivity. Central estimates for all glaciers show a maximum difference of only 8%. That means if mineral dust and BC contributed 5% to snow melt with 1% Fe mineral dust concentration, it would be 5.4% in the higher absorptivity case.

References

[1] M. Barandun, M. Huss, L. Sold, D. Farinotti, E. Azisov, N. Salzmann, R. Usubaliev, A. Merkushkin, and M. Hoelzle. Re-analysis of seasonal mass balance at Abramov Glacier 1968-2014. *Journal of Glaciology*, 61(230):1103–1117, accepted. doi: 10.3189/2015JoG14J239.

[2] M. Kronenberg, M. Barandun, M. Hoelzle, M. Huss, D. Farinotti, E. Azisov, R. Usubaliev, A. Gafurov, D. Petrakov, and A. Kääb. Mass balance reconstruction for Glacier No. 354, Tien Shan, from 2003-2014. *Annals of Glaciology*, 57(71):92–102, 2016. doi: 10.3189/201AoG71A032.

[3] D. Farinotti, L. Longuevergne, G. Moholdt, D. Duethmann, T. Mölg, T. Bolch, S. Vorogushyn, and A. Gϋtner. Substantial glacier mass loss in the Tien Shan over the past 50 years. *Nature Geoscience*, 8(9):716–722, 2015. doi: 10.1038/NGEO2513.

[4] EPA. Method 1669 Sampling Ambient Water for Trace Metals at EPA Water Quality Criteria Levels, 1996.

[5] J. Ming, H. Cachier, C. Xiao, D. Qin, S. Kang, S. Hou, and J. Xu. Black carbon record based on a shallow Himalayan ice core and its climatic implications. *Atmos. Chem. Phys.*, 8(5):1343–1352, 2008.

[6] H. Cachier and M. H. Pertuisot. Particulate carbon in Arctic ice. *Analusis,*, 22:M34–M37, 1994.

[7] P. Chýlek, V. Srivastava, L. Cahenzli, R.G Pinnick, R.L. Dod, T. Novakov, T.L. Cook, and B.D. Hinds. Aerosol and graphitic carbon content of snow. *Journal of Geophysical Research: Atmospheres (1984-2012)*, 92(D8):9801–9809, 1987.

[8] V.M.H Lavanchy, H.W. Gäggeler, U. Schotterer, M. Schwikowski, and U. Baltensperger. Historical record of carbonaceous particle concentrations from a European highalpine glacier (Colle Gnifetti, Switzerland). *Journal of Geophysical Research: Atmospheres (19842012)*, 104(D17):21227–21236, 1999.

[9] N. Dewan, B. J. Majestic, M. E. Ketterer, J. P. Miller-Schulze, M. M. Shafer, J. J. Schauer, P. A. Solomon, M. Artamonova, B. B. Chen, S. A. Imashev, and G. R. Carmichael. Stable isotopes of lead and strontium as tracers of sources of airborne particulate matter in Kyrgyzstan. *Atmospheric Environment*, 120:438–446, 2015.

[10] C. Uglietti, P. Gabrielli, J. W. Olesik, A. Lutton, and L. G. Thompson. Large variability of trace element mass fractions determined by ICP-SFMS in ice core samples from worldwide high altitude glaciers. *Applied Geochemistry*, 47:109–121, 2014.

[11] M. Sand, T. K. Berntsen, K. von Salzen, M. G. Flanner, J. Langner, and D. G. Victor. Response of Arctic temperature to changes in emissions of short-lived climate forcers. *Nature Clim. Change*, advance online publication, 2015.

[12] A. Stohl, B. Aamaas, M. Amann, L. H. Baker, N. Bellouin, T. K. Berntsen, O. Boucher, R. Cherian, W. Collins, N. Daskalakis, M. Dusinska, S. Eckhardt, J. S. Fuglestvedt, M. Harju, C. Heyes, Hodnebrog, J. Hao, U. Im, M. Kanakidou, Z. Klimont, K. Kupiainen, K. S. Law, M. T. Lund, R. Maas, C. R. MacIntosh, G. Myhre, S. Myriokefalitakis, D. Olivi, J. Quaas, B. Quennehen, J. C. Raut, S. T. Rumbold, B. H. Samset, M. Schulz, Seland, K. P. Shine, R. B. Skeie, S. Wang, K. E. Yttri, and T. Zhu. Evaluating the climate

and air quality impacts of short-lived pollutants. *Atmos. Chem. Phys.*, 15(18):10529–10566, 2015.

[13] G. R. van der Werf, J. T. Randerson, L. Giglio, G. J. Collatz, M. Mu, P. S. Kasibhatla, D. C. Morton, R. S. DeFries, Y. Jin, and T. T. van Leeuwen. Global fire emissions and the contribution of deforestation, savanna, forest, agricultural, and peat fires (19972009). *Atmos. Chem. Phys.*, 10(23):11707–11735, 2010.

[14] V. B. Aizen, P. A. Mayewski, E. M. Aizen, D. R. Joswiak, A. B. Surazakov, S. Kaspari, B. Grigholm, M. Krachler, M. Handley, and A. Finaev. Stable-isotope and trace element time series from Fedchenko glacier (Pamirs) snow/firn cores. *Journal of Glaciology*, 55(190):275–291, 2009.

[15] R. Kulmatov and M. Hojamberdiev. Distribution of heavy metals in atmospheric air of the arid zones in Central Asia. *Air Quality, Atmosphere & Health*, 3(4):183–194, 2010.

[16] E. Journet, Y. Balkanski, and S. P. Harrison. A new data set of soil mineralogy for dust-cycle modeling. *Atmos. Chem. Phys.*, 14(8):3801–3816, 2014.

[17] X.L. Zhang, G.J. Wu, C.L. Zhang, T.L. Xu, and Q.Q. Zhou. What is the real role of iron oxides in the optical properties of dust aerosols? *Atmospheric Chemistry and Physics*, 15(21):12159–12177, 2015.

[18] J. Huang, Q. Fu, W. Zhang, X. Wang, R. Zhang, H. Ye, and S. G. Warren. Dust and black carbon in seasonal snow across Northern China. *Bulletin of the American Meteorological Society*, 92(2):175–181, 2011.

[19] S. Kaspari, I. Skiles, M. Delaney, D. Dixon, and T.H. Painter. Accelerated glacier melt on Snow Dome, Mount Olympus, Washington, USA, due to deposition of black carbon and mineral dust from wildfire. *Journal of Geophysical Research: Atmospheres*, 120(7):2793–2807, 2015.

[20] S. J. Doherty, T. C. Grenfell, S. Forsstrm, D. L. Hegg, R. E. Brandt, and S. G. Warren. Observed vertical redistribution of black carbon and other insoluble light-absorbing particles in melting snow. *Journal of Geophysical Research D: Atmospheres*, 118(11):5553–5569, 2013.

[21] J. Oerlemans and E.J Klok. Energy balance of a glacier surface: Analysis of automatic weather station data from the moratschgletscher, switzerland. *Arctic, Antarctic and Alpine Research*, 34:477–485, 2002.

[22] P. Gabrielli, A. Wegner, J. R. Petit, B. Delmonte, P. De Deckker, V. Gaspari, H. Fischer, U. Ruth, M. Kriews, C. Boutron, P. Cescon, and C. Barbante. A major glacial-interglacial change in aeolian dust composition inferred from Rare Earth Elements in Antarctic ice. *Quaternary Science Reviews*, 29(12):265–273, 2010.

[23] V. Gaspari, C. Barbante, G. Cozzi, P. Cescon, C. F. Boutron, P. Gabrielli, G. Capodaglio, C. Ferrari, J. R. Petit, and B. Delmonte. Atmospheric iron fluxes over the last deglaciation: Climatic implications. *Geophysical Research Letters*, 33(3):L03704, 2006.

[24] U. Ruth, C. Barbante, M. Bigler, B. Delmonte, H. Fischer, P. Gabrielli, V. Gaspari, P. Kaufmann, F. Lambert, V. Maggi, F. Marino, J.R. Petit, R. Udisti, D. Wagenbach, A. Wegner, and E. W. Wolff. Proxies and Measurement Techniques for Mineral Dust in Antarctic Ice Cores. *Environmental Science & Technology*, 42(15):5675–5681, 2008.

[25] H.K. Wedepohl. The composition of the continental crust. *Geochimica et Cosmochimica Acta*, 59(7):1217–1232, 1995.
